# Supplementary material for: Efficacy of trimetazidine for myocardial ischemia-reperfusion injury in rat models: a systematic review and meta-analysis
Source: PeerJ. 2025 Jun 6;13:e19515. doi: 10.7717/peerj.19515 (PMC12147767; doi:10.7717/peerj.19515)
Supplement: Supplemental Information 1 [file peerj-13-19515-s001.docx]

**Supplementary Figures 1-36**


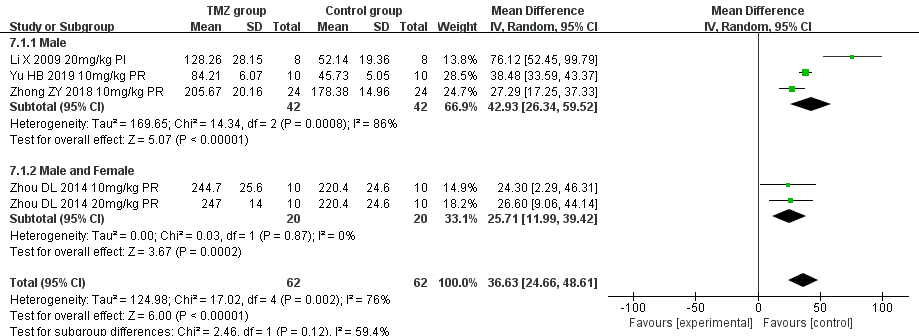


Supplementary Figure 1. Subgroup analysis of SOD based on gender distribution.


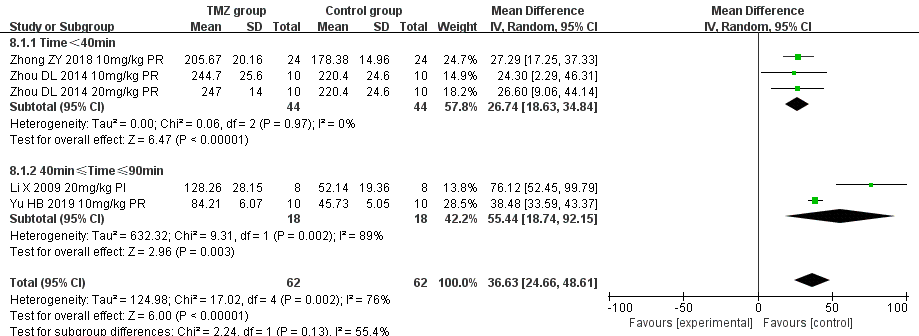


Supplementary Figure 2. Subgroup analysis of SOD based on ischemia duration.


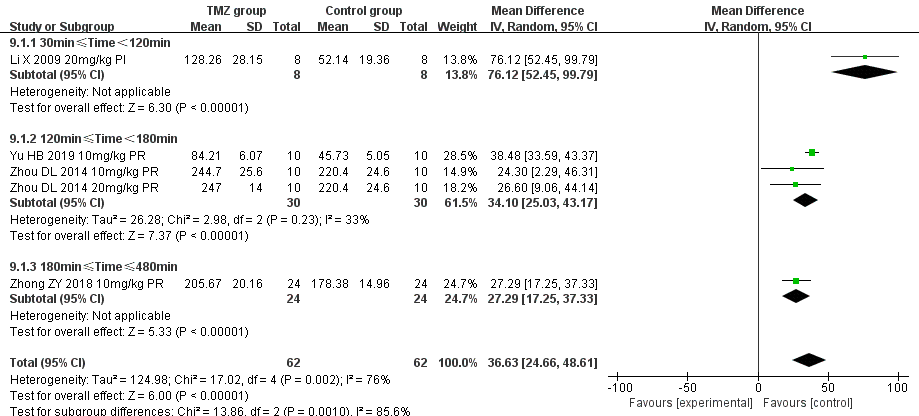


Supplementary Figure 3. Subgroup analysis of SOD based on reperfusion duration.


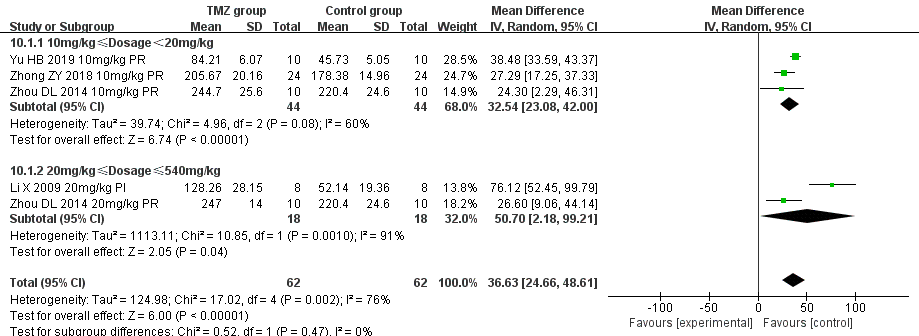


Supplementary Figure 4. Subgroup analysis of SOD based on dosage.


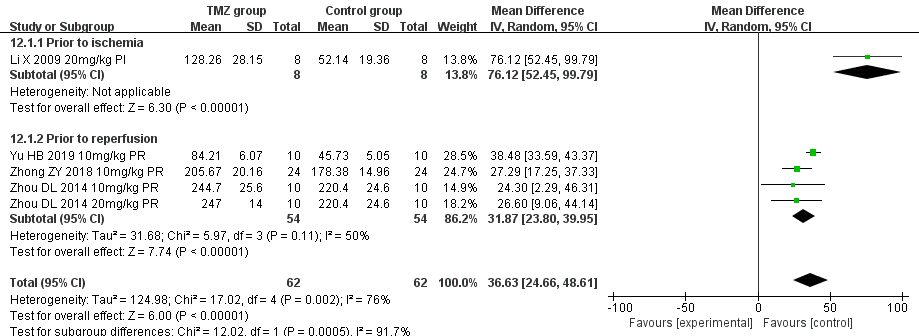


Supplementary Figure 5. Subgroup analysis of SOD based on treatment time.


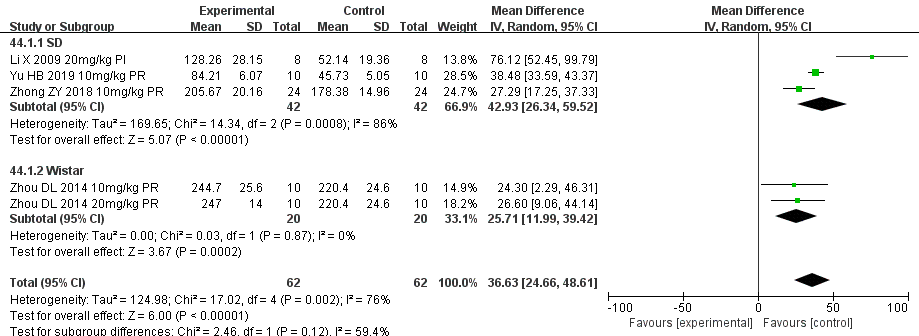


Supplementary Figure 6. Subgroup analysis of SOD based on rat species.


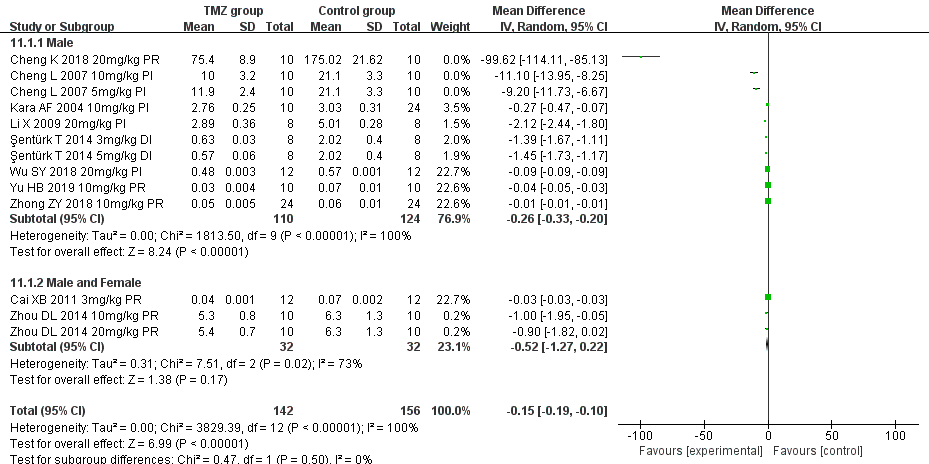


Supplementary Figure 7. Subgroup analysis of MDA based on gender distribution.


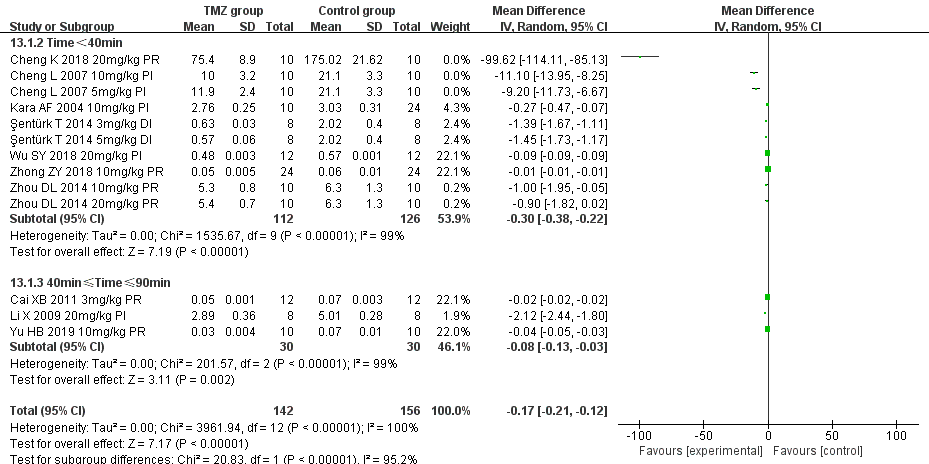


Supplementary Figure 8. Subgroup analysis of MDA based on ischemia duration.


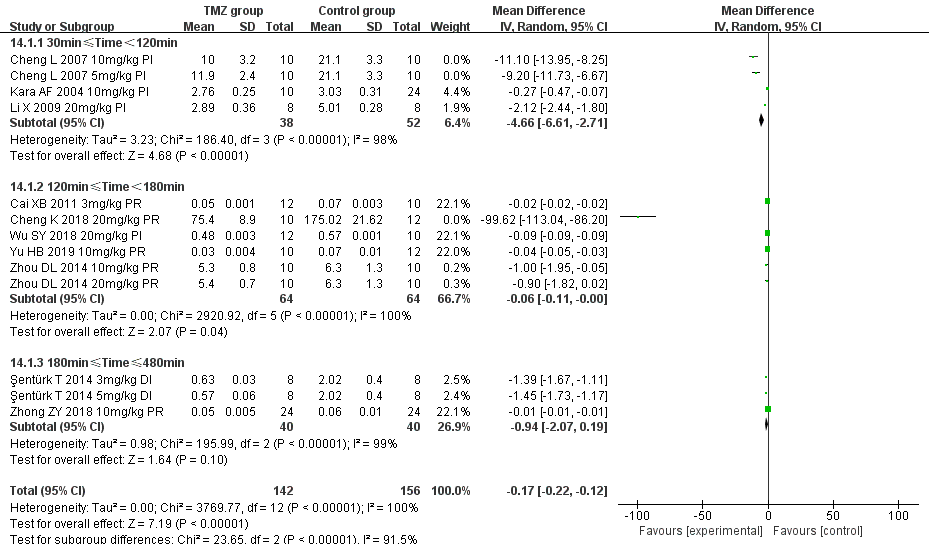


Supplementary Figure 9. Subgroup analysis of MDA based on reperfusion duration.


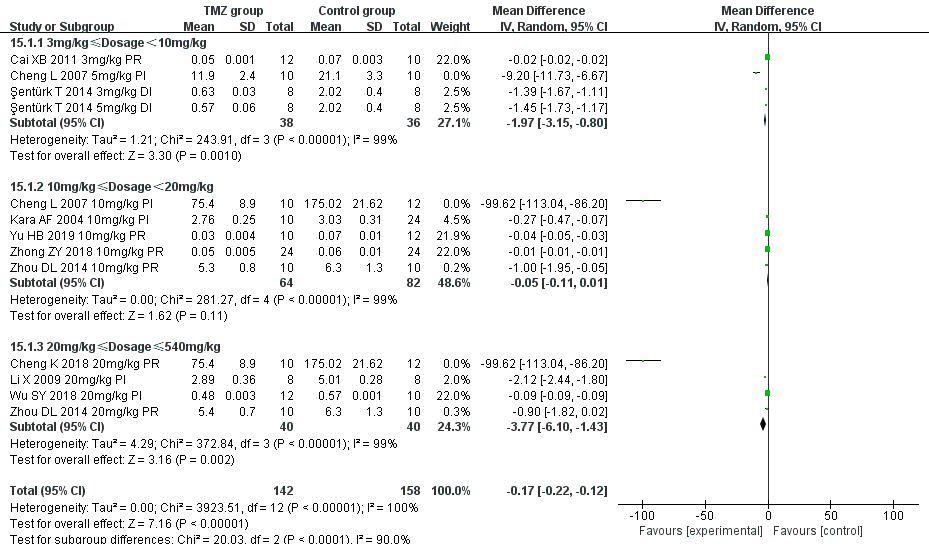


Supplementary Figure 10. Subgroup analysis of MDA based on dosage.


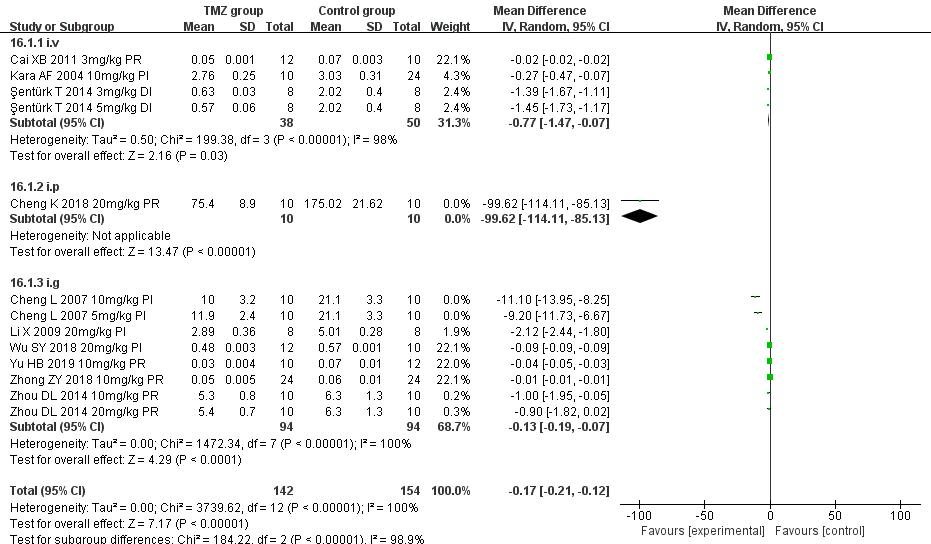


Supplementary Figure 11. Subgroup analysis of MDA based on routes.


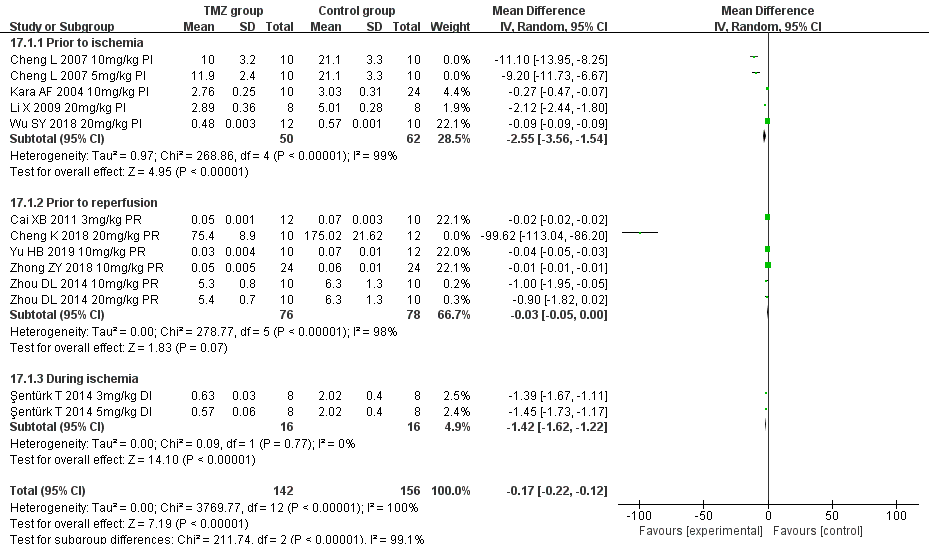


Supplementary Figure 12. Subgroup analysis of MDA based on treatment time.


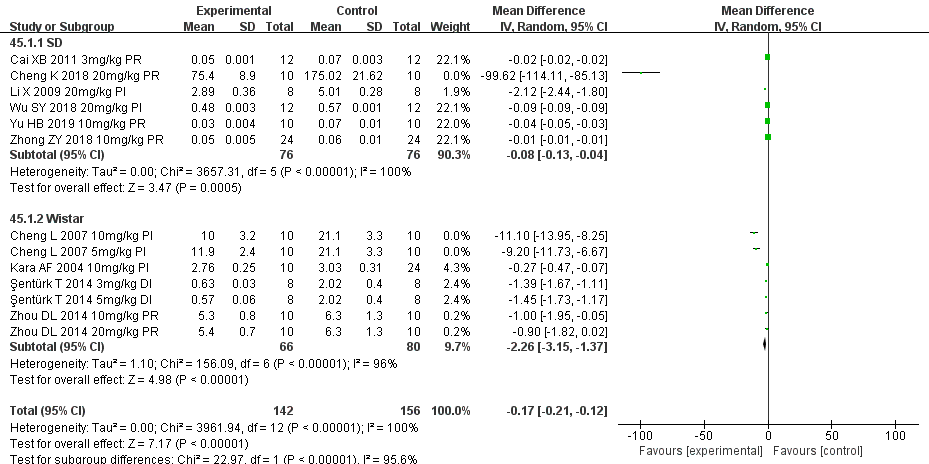


Supplementary Figure 13. Subgroup analysis of MDA based on rat species.


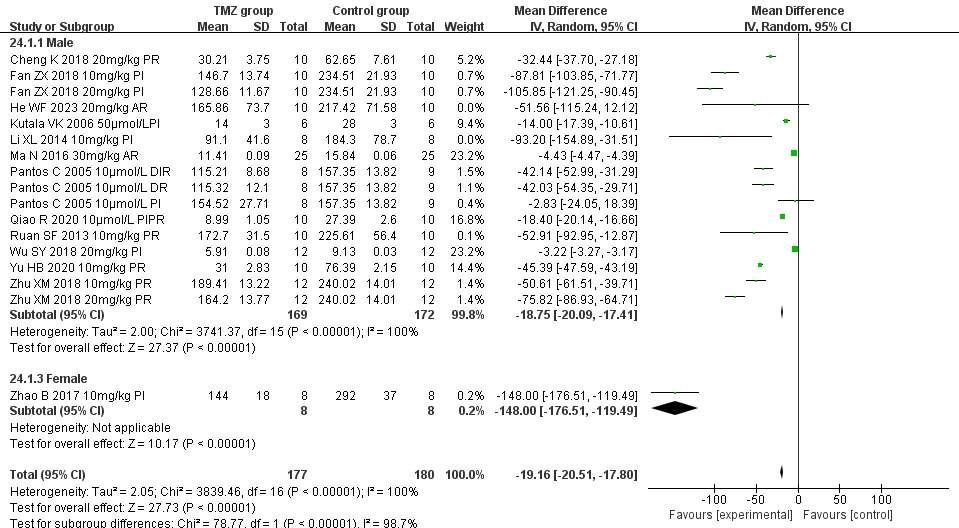


Supplementary Figure 14. Subgroup analysis of LDH based on gender distribution.


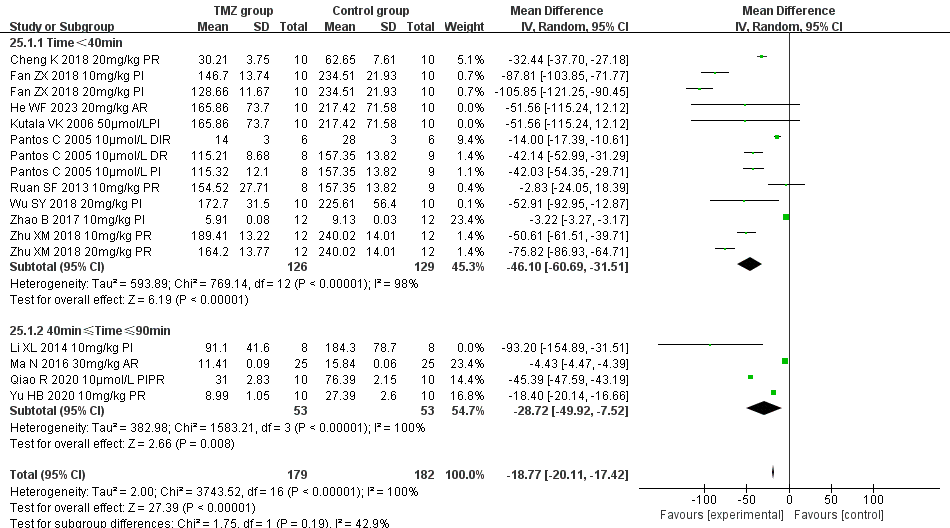


Supplementary Figure 15. Subgroup analysis of LDH based on ischemia duration.


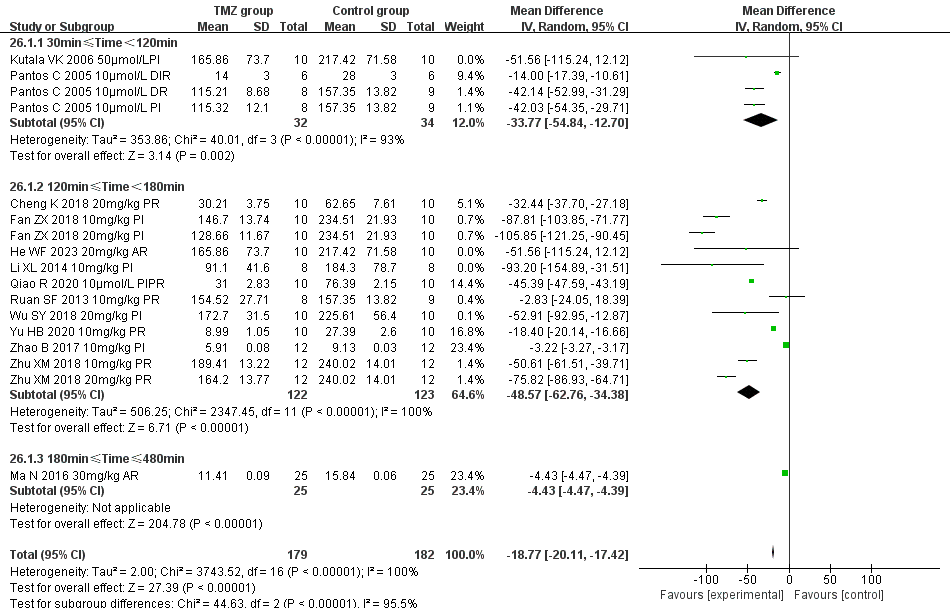


Supplementary Figure 16. Subgroup analysis of LDH based on reperfusion duration.


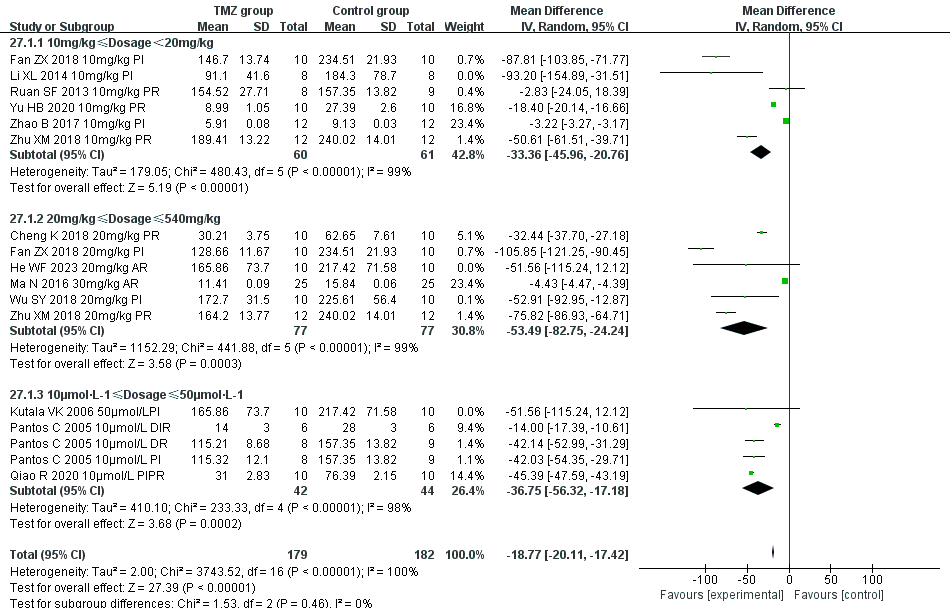


Supplementary Figure 17. Subgroup analysis of LDH based on dosage.


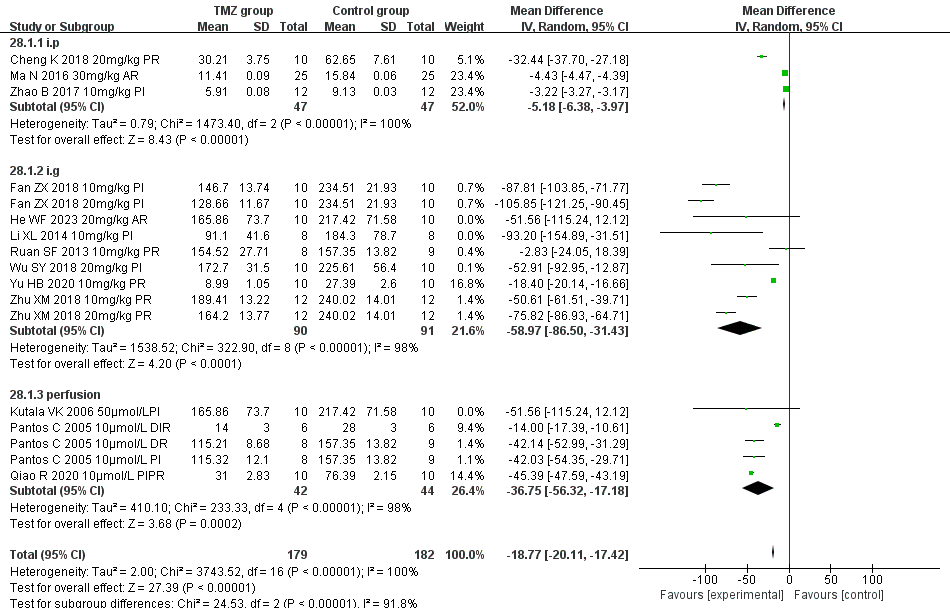


Supplementary Figure 18. Subgroup analysis of LDH based on route.


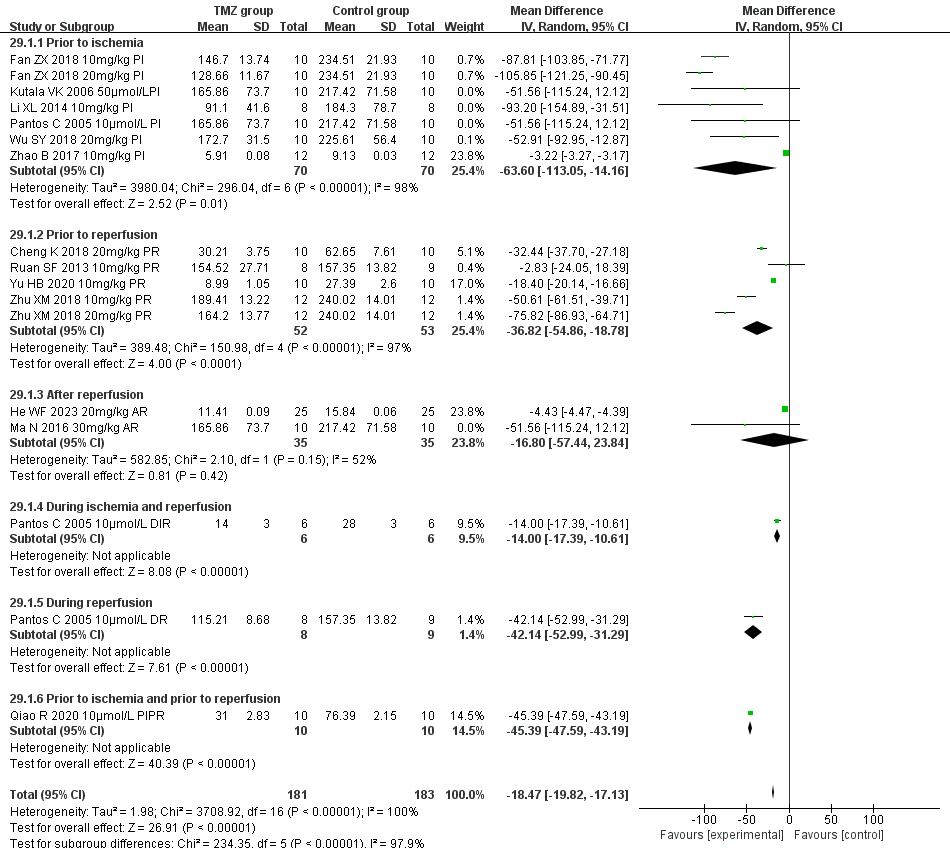


Supplementary Figure 19. Subgroup analysis of LDH based on treatment time.


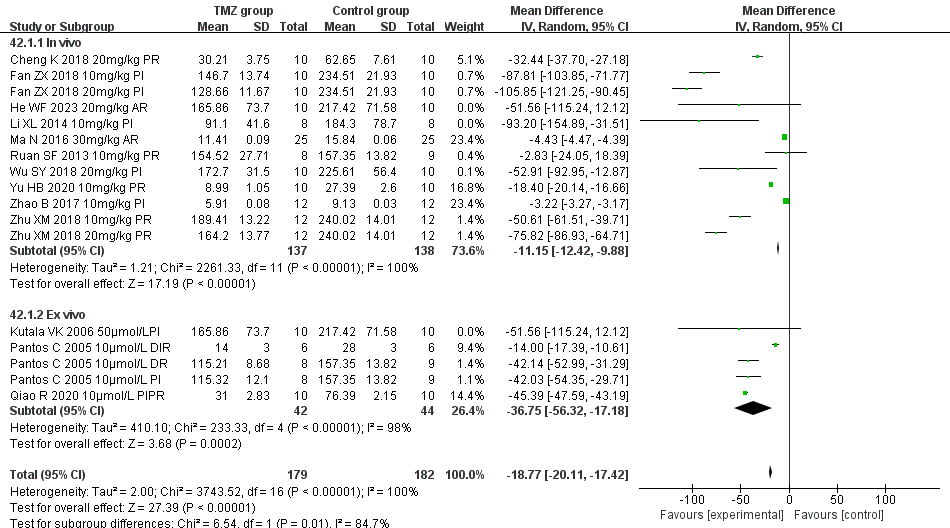


Supplementary Figure 20 . Subgroup analysis of LDH based on experiment type.


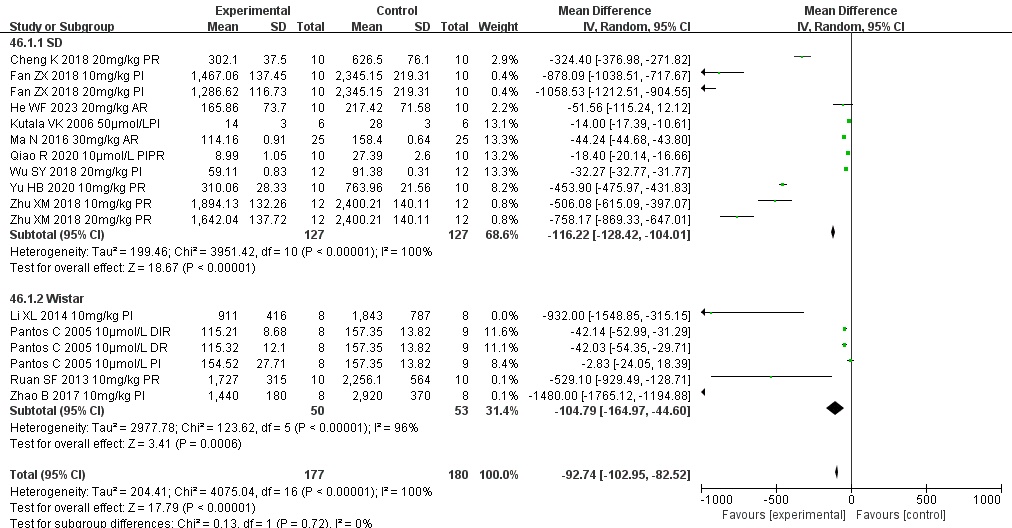


Supplementary Figure 21. Subgroup analysis of LDH based on rat species.


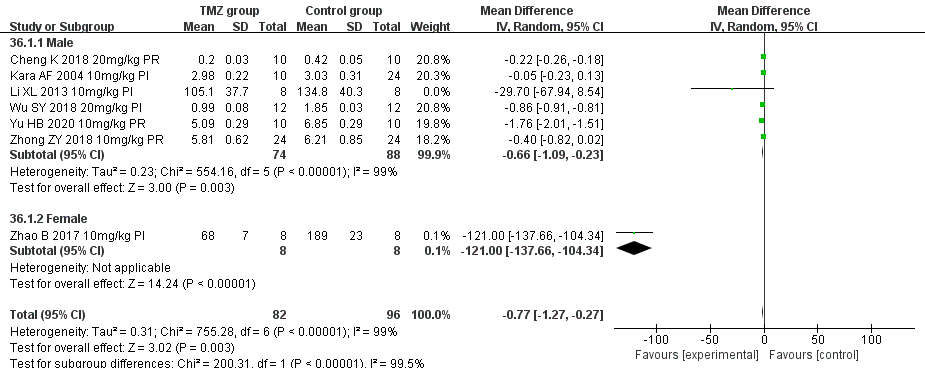


Supplementary Figure 22. Subgroup analysis of CK-MB based on gender distribution.


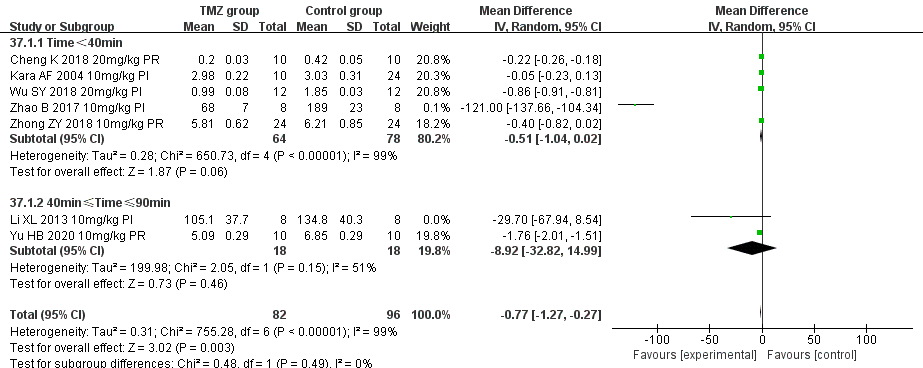


Supplementary Figure 23. Subgroup analysis of CK-MB based on ischemia duration.


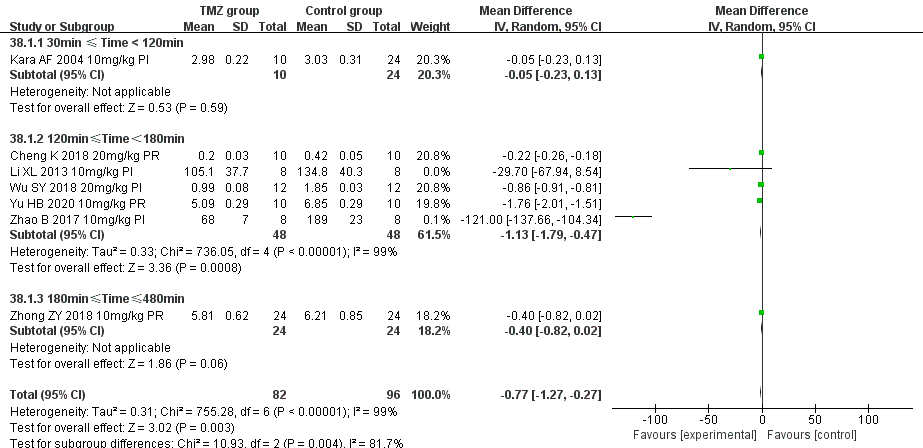


Supplementary Figure 24. Subgroup analysis of CK-MB based on reperfusion duration.


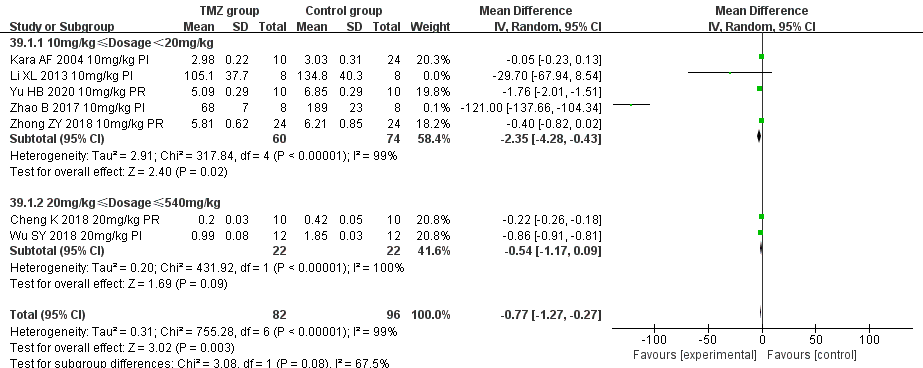


Supplementary Figure 25. Subgroup analysis of CK-MB based on dosage.


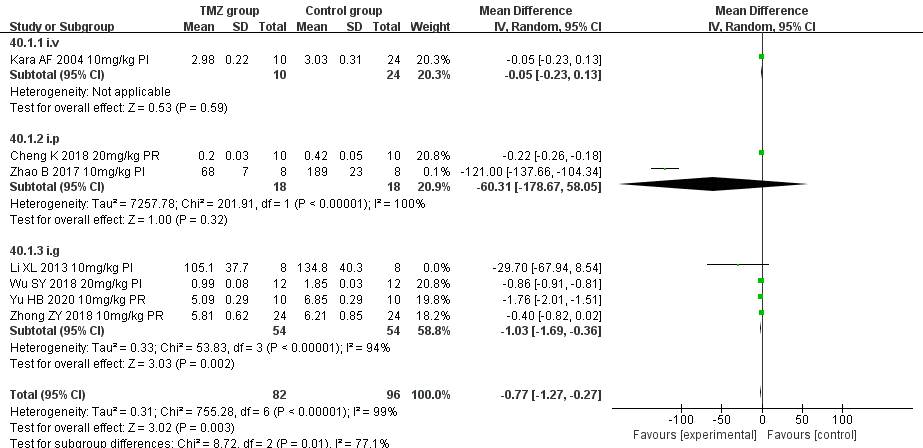


Supplementary Figure 26. Subgroup analysis of CK-MB based on route.


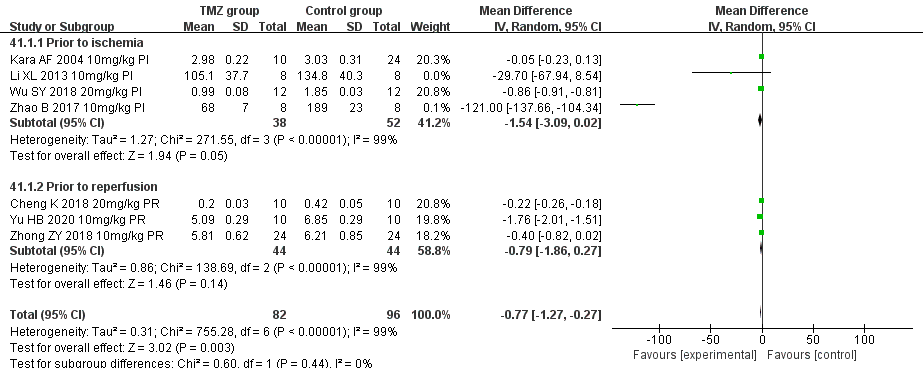


Supplementary Figure 27. Subgroup analysis of CK-MB based on treatment time.


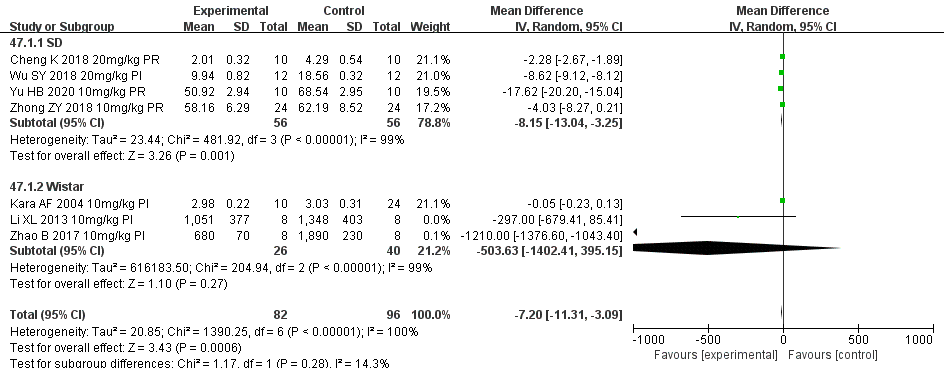


Supplementary Figure 28. Subgroup analysis of CK-MB based on rat species.


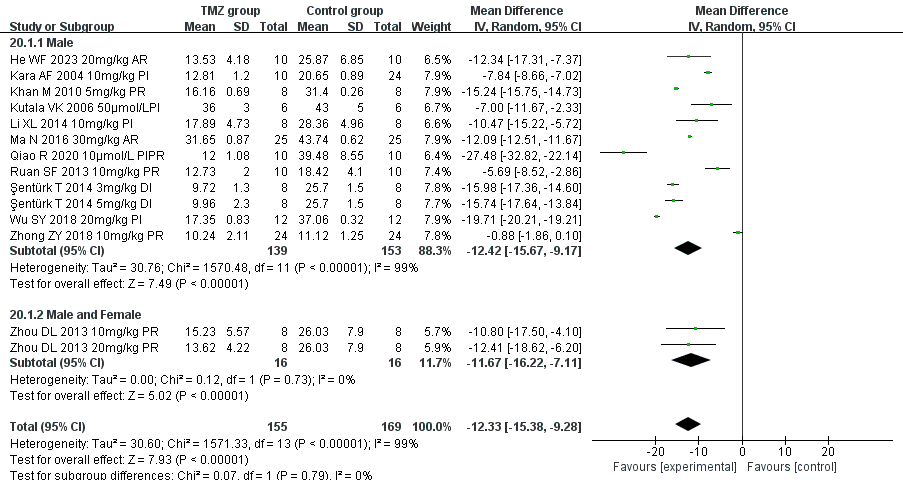


Supplementary Figure 29. Subgroup analysis of myocardial infarct size based on gender distribution.


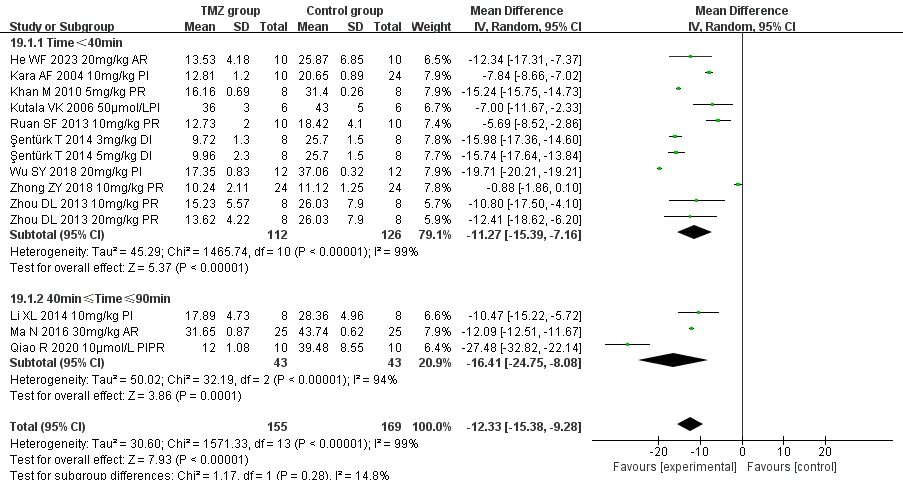


Supplementary Figure 30. Subgroup analysis of myocardial infarct size based on ischemia duration.


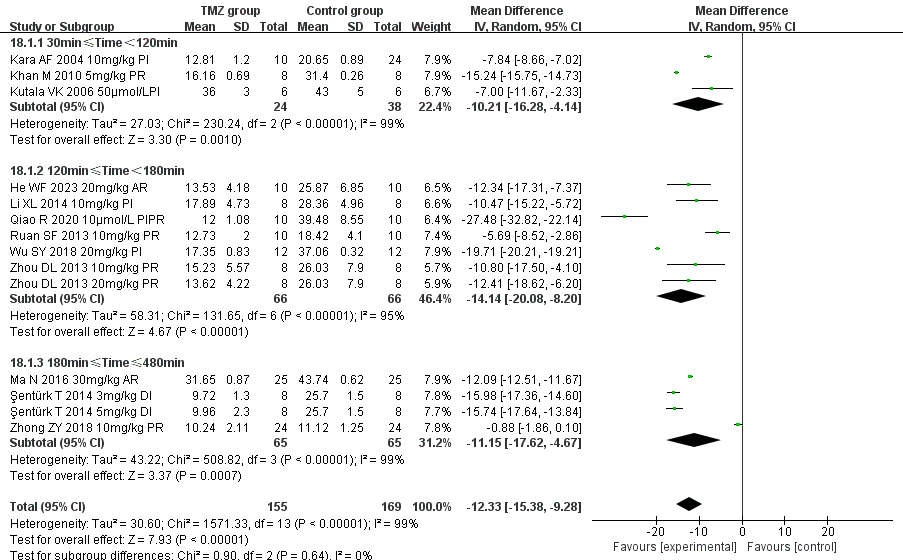


Supplementary Figure 31. Subgroup analysis of myocardial infarct size based on reperfusion duration.


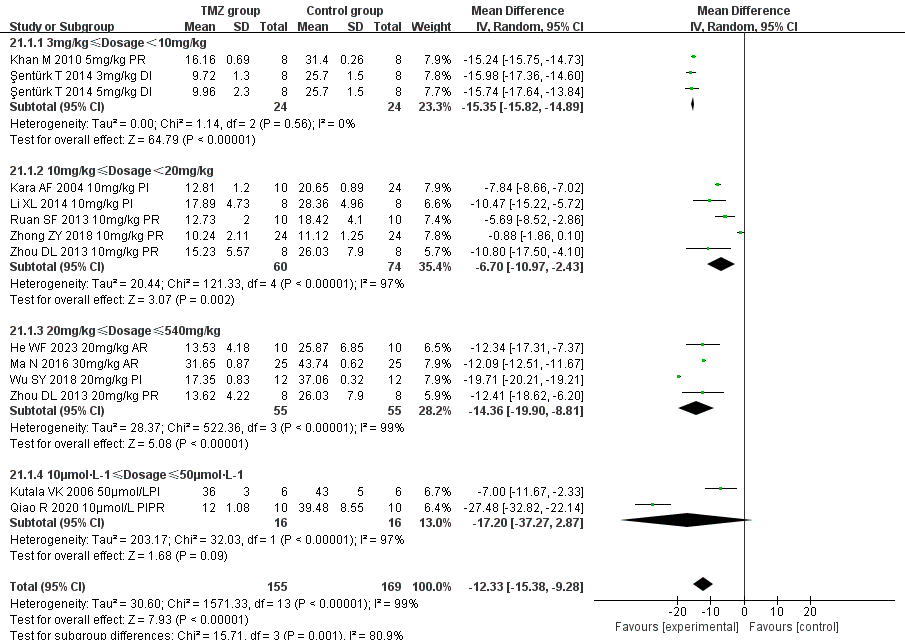


Supplementary Figure 32. Subgroup analysis of myocardial infarct size based on dosage.


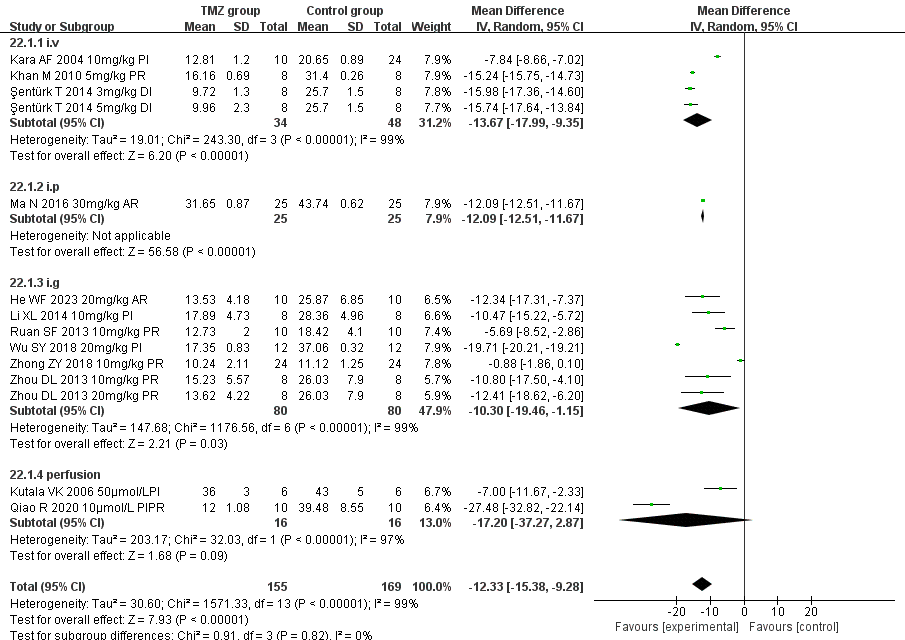


Supplementary Figure 33. Subgroup analysis of myocardial infarct size based on route.


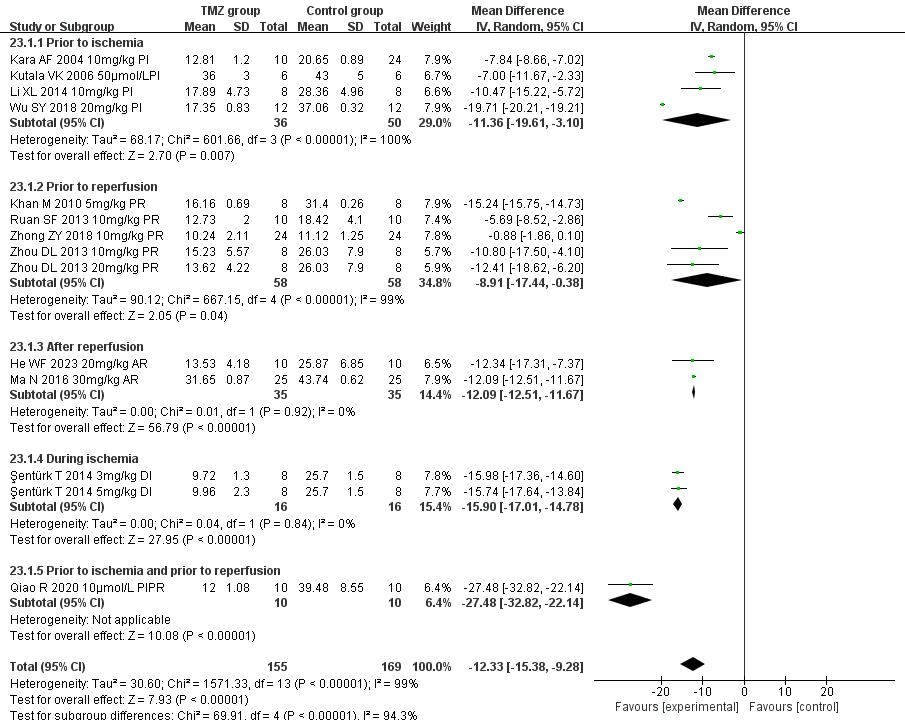


Supplementary Figure 34. Subgroup analysis of myocardial infarct size based on treatment time


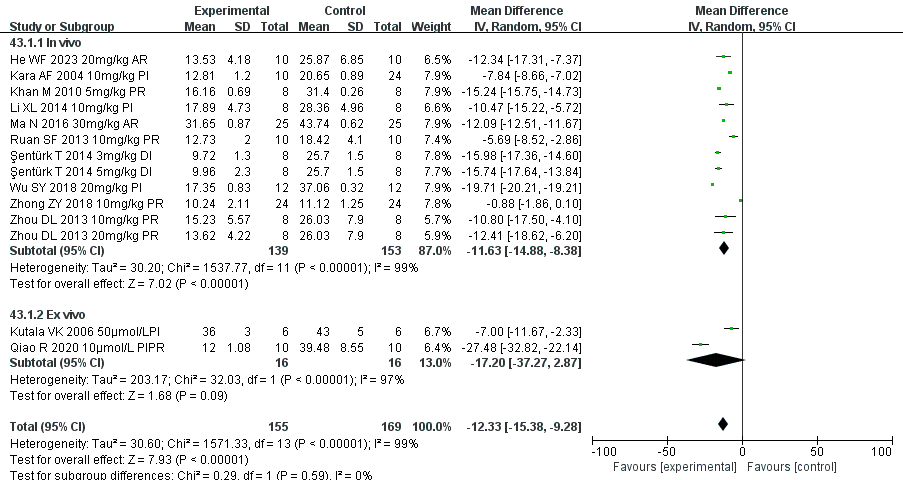


Supplementary Figure 35. Subgroup analysis of myocardial infarct size based on experiment type.


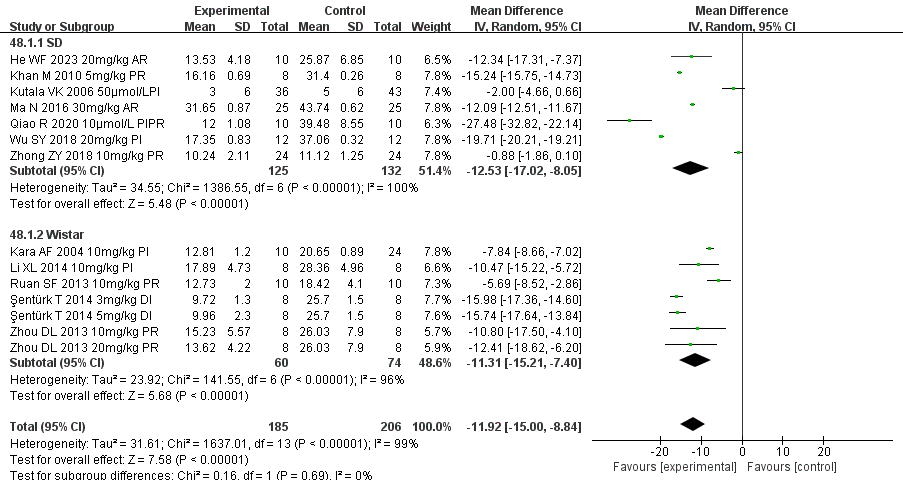


Supplementary Figure 36. Subgroup analysis of myocardial infarct size based on rat species.
