# Supplementary material for: Efficacy of trimetazidine for myocardial ischemia-reperfusion injury in rat models: a systematic review and meta-analysis
Source: PeerJ. 2025 Jun 6;13:e19515. doi: 10.7717/peerj.19515 (PMC12147767; doi:10.7717/peerj.19515)
Supplement: Supplemental Information 2 [file peerj-13-19515-s002.docx]

**Table S1. Detailed database search strategy**

**English database**

**Pubmed: 34**

((((((((((("Myocardial Reperfusion Injury"[Mesh]) OR (Myocardial reperfusion Injury[Title/Abstract])) OR (Injuries, Myocardial Reperfusion[Title/Abstract])) OR (Myocardial Reperfusion Injuries[Title/Abstract])) OR (Reperfusion Injuries, Myocardial[Title/Abstract])) OR (Reperfusion Injury, Myocardial[Title/Abstract])) OR (Injury, Myocardial Reperfusion[Title/Abstract])) OR (Myocardial Ischemic Reperfusion Injury[Title/Abstract])) OR (Myocardial ischemia-reperfusion injury[Title/Abstract])) OR (Myocardial ischemia reperfusion injury[Title/Abstract])) AND (((((((((("Rats"[Mesh]) OR (Rats[Title/Abstract])) OR (Rat[Title/Abstract])) OR (Rattus[Title/Abstract])) OR (Rattus norvegicus[Title/Abstract])) OR (Rats, Norway[Title/Abstract])) OR (Rats, Laboratory[Title/Abstract])) OR (Laboratory Rat[Title/Abstract])) OR (Laboratory Rats[Title/Abstract])) OR (Rat, Laboratory[Title/Abstract]))) AND (((((((((("Trimetazidine"[Mesh]) OR (Trimetazidine[Title/Abstract])) OR (TMZ[Title/Abstract])) OR (Centrophène[Title/Abstract])) OR (Trimetazidine Dihydrochloride[Title/Abstract])) OR (Dihydrochloride, Trimetazidine[Title/Abstract])) OR (Vastarel[Title/Abstract])) OR (Trimétazidine Irex[Title/Abstract])) OR (Vasartel[Title/Abstract])) OR (Idaptan[Title/Abstract]))

**Web of Science：49**

(TS=(Myocardial Reperfusion Injury) OR AB=(Myocardial reperfusion Injury OR Injuries, Myocardial Reperfusion OR Myocardial Reperfusion Injuries OR Reperfusion Injuries, Myocardial OR Reperfusion Injury, Myocardial OR Injury, Myocardial Reperfusion OR Myocardial Ischemic Reperfusion Injury OR Myocardial ischemia-reperfusion injury OR Myocardial ischemia reperfusion injury)) AND (TS=(Rats) OR AB=(Rats OR Rat OR Rattus OR Rattus norvegicus OR Rats, Norway OR Rats, Laboratory OR Laboratory Rat OR Laboratory Rats OR Rat, Laboratory)) AND (TS=(Trimetazidine) OR AB=(Trimetazidine OR TMZ OR Centrophène OR Trimetazidine Dihydrochloride OR Dihydrochloride, Trimetazidine OR Vastarel OR Trimétazidine Irex OR Vasartel OR Idaptan))

**Embase:14**

Embase

Session Results

.......................................................

No. Query Results Results Date

#4. #1 AND #2 AND #3 14 30 Jun 2024

#3. trimetazidine:ab,ti OR tmz:ab,ti OR 9,030 30 Jun 2024

centrophène:ab,ti OR 'trimetazidine

dihydrochloride':ab,ti OR 'dihydrochloride,

trimetazidine':ab,ti OR vastarel:ab,ti OR

'trimétazidine irex':ab,ti OR vasartel:ab,ti OR

idaptan:ab,ti

#2. rats:ab,ti OR rat:ab,ti OR rattus:ab,ti OR 1,735,335 30 Jun 2024

'rattus norvegicus':ab,ti OR 'rats, norway':ab,ti

OR 'rats, laboratory':ab,ti OR 'laboratory

rat':ab,ti OR 'laboratory rats':ab,ti OR 'rat,

laboratory':ab,ti

#1. 'myocardial reperfusion injury':ab,ti OR 5,756 30 Jun 2024

'injuries, myocardial reperfusion':ab,ti OR

'myocardial reperfusion injuries':ab,ti OR

'reperfusion injuries, myocardial':ab,ti OR

'reperfusion injury, myocardial':ab,ti OR

'injury, myocardial reperfusion':ab,ti OR

'myocardial ischemic reperfusion injury':ab,ti OR

'myocardial ischemia-reperfusion injury':ab,ti OR

'myocardial ischemia reperfusion injury':ab,ti

.......................................................

**Cochrane Library:0**

Search Name: Date Run: 30/06/2024 15:44:49

Comment:

ID Search Hits

#1 MeSH descriptor: [Myocardial Reperfusion Injury] explode all trees 559

#2 (Myocardial reperfusion Injury or Injuries, Myocardial Reperfusion or Myocardial Reperfusion Injuries or Reperfusion Injuries, Myocardial or Reperfusion Injury, Myocardial or Injury, Myocardial Reperfusion or Myocardial Ischemic Reperfusion Injury or Myocardial ischemia-reperfusion injury or Myocardial ischemia reperfusion injury):ti,ab,kw (Word variations have been searched) 1658

#3 #1 or #2 1658

#4 MeSH descriptor: [Rats] explode all trees 1124

#5 (Rats or Rat or Rattus or Rattus norvegicus or Rats, Norway or Rats, Laboratory or Laboratory Rat or Laboratory Rats or Rat, Laboratory):ti,ab,kw (Word variations have been searched) 4492

#6 #4 or #5 4492

#7 MeSH descriptor: [Trimetazidine] explode all trees 216

#8 (Trimetazidine or TMZ or Centrophène or Trimetazidine Dihydrochloride or Dihydrochloride, Trimetazidine or Vastarel or Trimétazidine Irex or Vasartel or Idaptan):ti,ab,kw (Word variations have been searched) 1178

#9 #7 or #8 1178

#10 #3 and #6 and #9 0

**Chinese database**

**China National KnowledgeInfrastructure database (CNKI) (in the advanced search): 93**

(Subject: Myocardial ischemia-reperfusion injury (exact)) AND (Excerpt: Myocardial ischemia-reperfusion injury + Ischemia-reperfusion injury (exact)) AND (Subject: Rat (exact)) AND (Extract: Rat (exact)) AND (Subject: Trimetazidine (exact)) AND (Excerpt: Trimetazidine (exact))

**Wan-fang database (Wanfang) (in the advanced search): 79**

(subject: (myocardial ischemia-reperfusion injury) or title or keyword: (myocardial ischemia-reperfusion injury or ischemia-reperfusion injury)) and (subject: (rat) or title or keyword: (rat)) and (Subject: (Trimetazidine) or Title or Keyword: (Trimetazidine))

Chinese Scientific Journals database (VIP) **(in the advanced search): 84**

Title or keyword = myocardial ischemia-reperfusion injury or ischemia-reperfusion injury AND title or keyword = rat AND title or keyword = Trimetazidine

**Chinese Biomedicine database (CBM) (in the advanced search): 52**

("myocardial ischemia-reperfusion injury" [Common fields: Intelligent] OR "ischemia-reperfusion " [Common fields: Intelligent]) AND "rat" [Common fields: Intelligent] AND ("Trimetazidine" [Common fields: Intelligent] OR "TMZ" [Common fields: Intelligent])
