# Supplementary material for: Efficacy of trimetazidine for myocardial ischemia-reperfusion injury in rat models: a systematic review and meta-analysis
Source: PeerJ. 2025 Jun 6;13:e19515. doi: 10.7717/peerj.19515 (PMC12147767; doi:10.7717/peerj.19515)
Supplement: Supplemental Information 3 [file peerj-13-19515-s003.docx]

**TABLE S2.** Characteristics of the included studies.

| **Study** | **Species** | **Sex** | **Weight** | **Ischemia duration** | **Reperfusion duration** | **Groups(n)** | **Dosage** | **Route** | **Treatment time** | **Outcome**  **measure** |
| --- | --- | --- | --- | --- | --- | --- | --- | --- | --- | --- |
| Cai XB 2011 | SD | Male and Female | 200～250g | 90min | 120min | Control group (n= 12)  TMZ group (n= 12) | 0.9%NS 3mg·kg^-1^·d^-1^ | i.v | Prior to reperfusion | MDA |
|  |  |  |  |  |  |  | 3mg·kg^-1^·d^-1^ |  |  |  |
| Cheng K 2018 | SD | Male | 180~220g | 30min | 120min | Control group (n= 10)  TMZ group (n= 10) | ?  20 mg·kg^-1^·d^-1^ | i.p | Prior to reperfusion | MDA, LDH, CK-MB |
| Li X  2009 | SD | Male | 150～180g | 60 min | 30min | Control group (n= 8)  TMZ group (n= 8) | PW 10 mL·kg^-1^·d^-1^  20 mg·kg^-1^·d^-1^ | i.g | Prior to ischemia | SOD, MDA |
| Li XL 2013 | Wistar | Male | 230~280g | 40 min | 120min | Control group (n= 8)  TMZ group (n= 8) | 0.9%NS  10 mg·kg^-1^·d^-1^ | i.g | Prior to ischemia | CK-MB |
| Ruan SF 2013 | Wistar | Male | 230～270g | 30 min | 120min | Control group (n= 10)  TMZ group (n= 10) | ?  10 mg·kg^-1^·d^-1^ | i.g | Prior to reperfusion | LDH, MIS |
| Yu HB  2019 | SD | Male | 280~300g | 45min | 120 min | Control group (n= 10)  TMZ group (n= 10) | 0.9%NS  10 mg·kg^-1^·d^-1^ | i.g | Prior to reperfusion | SOD, MDA |
| Zhao B  2017 | Wistar | Female | 200~250g | 30min | 120 min | Control group (n= 8)  TMZ group (n= 8) | 0.9%NS 10 mg·kg^-1^·d^-1^  10 mg·kg^-1^·d^-1^ | i.p | Prior to ischemia | LDH, CK-MB |
| Zhong ZY 2018 | SD | Male | 220～280g | 30 min | 480min | Control group (n= 24)  TMZ group (n= 24) | 0.9%NS  10 mg·kg^-1^·d^-1^ | i.g | Prior to reperfusion | SOD, MDA, CK-MB, MIS |
| Yu HB 2020 | SD | Male | 280～300g | 45min | 120 min | Control group (n= 10)  TMZ group (n= 10) | 0.9%NS  10 mg·kg^-1^·d^-1^ | i.g | Prior to reperfusion | LDH, CK-MB |
| Zhu XM 2018 | SD | Male | 250~300g | 30 min | 120 min | Control group (n= 12)  TMZ-L group (n= 12)  TMZ-H group (n= 12) | 0.9%NS  10 mg·kg^-1^·d^-1^  20 mg·kg^-1^·d^-1^ | i.g | Prior to reperfusion | LDH |
| Li XL  2014 | Wistar | Male | 230~280g | 40 min | 120 min | Control group (n= 8)  TMZ group (n= 8) | 0.9%NS  10mg·kg^-1^·d^-1^ | i.g | Prior to ischemia | LDH, MIS |
| Ma N  2016 | SD | Male | 250~300 g | 40 min | 180 min | Control group (n= 25)  TMZ group (n= 25) | 0.9%NS 0.1mL·100g^-1^·d^-1^  30mg·kg^-1^·d^-1^ | i.p | After reperfusion | LDH, MIS |
| Wu SY  2018 | SD | Male | 160~240g | 30 min | 120 min | Control group (n= 12)  TMZ group (n= 12) | 0.9%NS 5ml·kg^-1^·d^-1^  20mg·kg^-1^·d^-1^ | i.g | Prior to ischemia | MDA, LDH, CK-MB, MIS |
| (Continued on the following page) | | | | | | | | | | |
| **TABLE S2.** (*Continued*) Characteristics of the included studies. | | | | | | | | | | |
| **Study** | **Species** | **Sex** | **Weight** | **Ischemia duration** | **Reperfusion duration** | **Groups(n)** | **Dosage** | **Route** | **Treatment time** | **Outcome**  **measure** |
| Zhou DL 2014 | Wistar | Male and Female | 180~220g | 30 min | 120 min | Control group (n= 10)  TMZ-L group (n= 10)  TMZ-H group (n= 10) | 0.9%NS  10 mg·kg^-1^·d^-1^  20 mg·kg^-1^·d^-1^ | i.g | Prior to reperfusion | SOD, MDA |
| Cheng L 2007 | Wistar | Male | 200~250g | 10 min | 30 min | Control group (n= 10)  TMZ-L group (n= 10)  TMZ-H group (n= 10) | 0.85%NS 2 mL·d^-1^  5 mg·kg^-1^·d^-1^  10 mg·kg^-1^·d^-1^ | i.g | Prior to ischemia | MDA |
| Fan ZX 2018 | SD | Male | 220～250g | 30 min | 120 min | Control group (n= 10)  TMZ-L group (n= 10)  TMZ-H group (n= 10) | ？  10mg·kg^-1^·d^-1^  20mg·kg^-1^·d^-1^ | i.g | Prior to ischemia | LDH |
| Zhou DL 2013 | Wistar | Male and Female | 180～220g | 30 min | 120 min | Control group (n= 8)  TMZ-L group (n= 8)  TMZ-H group (n= 8) | 0.9%NS  10 mg·kg-1·d-1  20 mg·kg-1·d-1 | i.g | Prior to reperfusion | MIS |
|  |  |  |  |  |  |  |  |  |  |  |
| Khan M 2010 | SD | Male | 300 ~ 350g | 30 min | 60 min | Control group (n= 8)  TMZ group (n= 8) | 0.9%NS 0.5 ml·d^-1^  5 mg·kg^-1^·d^-1^ | i.v | Prior to reperfusion | MIS |
| Kutala VK 2006 | SD | Male | 300 ~ 350g | 30 min | 45 min | Control group (n= 6)  TMZ group (n= 6) | ？  50 μmol/L | ecp | Prior to ischemia | MIS， LDH |
| Pantos C 2005 | Wistar | Male | 280 ~ 330g | 20 min | 45 min | Control group (n= 9)  TMZ-STAB group (n= 8)  TMZ-STAB+REP group (n= 8)  TMZ-REP group (n= 8) | --  10μmol/L  10μmol/L  10μmol/L | ecp | Prior to ischemia  During ischemia and reperfusion  During reperfusion | LDH |
| Şentürk T 2014 | Wistar | Male | 250 ~ 300g | 30 min | 180 min | Control group (n= 8)  TMZ-L group (n= 8)  TMZ-H group (n= 8) | NS  3 mg·kg^-1^·d^-1^  5 mg·kg^-1^·d^-1^ | i.v | During ischemia | MIS, MDA |

(Continued on the following page)

**TABLE S2.** (*Continued*) Characteristics of the included studies.

| **Study** | **Species** | **Sex** | **Weight** | **Ischemia duration** | **Reperfusion duration** | **Groups(n)** | **Dosage** | **Route** | **Treatment time** | **Outcome**  **measure** |
| --- | --- | --- | --- | --- | --- | --- | --- | --- | --- | --- |
| Kara AF 2004 | Wistar | Male | 220 ~ 320 g | 5 min | 30 min | Control group (n= 24)  TMZ group (n= 10) | --  10 mg·kg^-1^·d^-1^ | i.v | Prior to ischemia | MIS, MDA，CK-MB |
| He WF 2023 | SD | Male | 200～250 g | 30 min | 120 min | Control group (n= 24)  TMZ group (n= 10) | NS 20 mg·kg^-1^·d^-1^  20 mg·kg^-1^·d^-1^ | i.g | After reperfusion | MIS, LDH |
| Qiao R 2020 | SD | Male | 220～250 g | 40 min | 120 min | Control group (n= 10)  TMZ group (n= 10) | --  10μmol/L | ecp | Prior to ischemia and prior to reperfusion | MIS, LDH |

*Abbreviations: TMZ, Trimetazidine; TMZ -L, Trimetazidine low-dose group; TMZ -H, Trimetazidine high-dose group; Control, receiving myocardial ischemic-reperfusion injury group;* *i.p, intraperitoneal injection; i.g, intragastric; i.v, intravenous injection; ecp, extracorporeal perfusion; NS, normal saline; PW, Purified water; SOD, superoxide dismutase; MDA, methane dicarboxylic aldehyde; LDH, lactic dehydrogenase; CK-MB, creatine kinase isoenzyme;* *MIS, myocardial infarct size; ?, not recorded.*
