# Supplementary material for: Efficacy of trimetazidine for myocardial ischemia-reperfusion injury in rat models: a systematic review and meta-analysis
Source: PeerJ. 2025 Jun 6;13:e19515. doi: 10.7717/peerj.19515 (PMC12147767; doi:10.7717/peerj.19515)
Supplement: Supplemental Information 4 [file peerj-13-19515-s004.docx]

**TABLE S3.** CAMARADES evaluation results.

| **Study** | **(A)** | **(B)** | **(C)** | **(D)** | **(E)** | **(F)** | **(G)** | **(H)** | **(I)** | **(J)** | **Total (Out of 10)** |
| --- | --- | --- | --- | --- | --- | --- | --- | --- | --- | --- | --- |
| Cai XB 2011 | * |  |  | * | * | * | * |  |  | * | 6 |
| Cheng K 2018 | * |  |  | * | * |  | * |  |  |  | 4 |
| Li X 2009 | * | * |  | * | * |  | * |  |  |  | 5 |
| Li XL 2013 | * |  |  | * | * | * | * |  |  |  | 5 |
| Ruan SF 2013 | * |  |  | * | * |  | * |  | * |  | 5 |
| Yu HB 2019 | * |  |  | * | * |  | * |  |  |  | 4 |
| Zhao B 2017 | * | * |  | * | * | * | * |  |  |  | 6 |
| Zhong ZY 2018 | * |  |  | * | * | * | * |  |  |  | 5 |
| Yu HB 2020 | * |  |  | * | * |  | * |  |  |  | 4 |
| Zhu XM 2018 | * |  |  | * | * | * | * | * |  |  | 6 |
| Li X 2014 | * | * |  | * | * |  | * |  |  | * | 6 |
| Ma N 2016 | * | * |  | * | * | * | * |  |  |  | 6 |
| Wu SY 2018 | * | * |  | * | * | * | * |  |  | * | 7 |
| Zhou DL 2014 | * |  |  | * | * | * | * | * |  |  | 6 |
| Cheng L 2007 | * |  |  | * | * | * | * | * |  |  | 6 |
| Fan ZX 2018 | * |  |  | * | * | * | * | * |  |  | 6 |
| Zhou DL 2013 | * |  |  | * | * |  | * | * |  |  | 5 |
| Khan M 2010 | * | * |  | * | * | * | * |  |  |  | 6 |
| Kutala VK 2006 | * |  |  | * | * |  | * |  |  |  | 4 |
| Pantos C 2005 | * | * |  | * | * | * | * |  |  |  | 6 |
| Şentürk T 2014 | * | * |  | * | * | * | * | * |  | * | 8 |
| Kara AF 2004 | * | * |  | * | * | * | * |  |  |  | 6 |
| He WF 2023 | * | * |  | * | * | * | * |  |  |  | 6 |
| Qiao R 2020 | * | * |  | * | * |  | * |  |  |  | 5 |

***(A)*** *Peer-reviewed publications;* ***(B)*** *statement of compliance with animal welfare regulations;* ***(C)*** *calculation of necessary sample size to achieve sufficient power;* ***(D)*** *randomization of subjects into treatment groups;* ***(E)*** *use of a suitable animal model;* ***(F)*** *monitoring of physiological parameters such as body temperature;* ***(G)*** *avoidance of anesthetic agents with marked affect cardiac function;* ***(H)*** *assessment of dose-response relationship;* ***(I)*** *blinded assessment of behavioral outcome;* ***(J)*** *statement of potential conflict of interests.*
