# Supplementary material for: Efficacy of trimetazidine for myocardial ischemia-reperfusion injury in rat models: a systematic review and meta-analysis
Source: PeerJ. 2025 Jun 6;13:e19515. doi: 10.7717/peerj.19515 (PMC12147767; doi:10.7717/peerj.19515)
Supplement: Supplemental Information 5 [file peerj-13-19515-s005.docx]

**TABLE S4.** Results of meta regression analysis.

| Meta regression analysis on the results of SOD | | | | | |
| --- | --- | --- | --- | --- | --- |
| _ES | Coefficient | Std. err | t | p＞\|t\| | [95% conf. interval] |
| Gender distribution | -2.67 | 1.17 | -2.28 | 0.056 | -5.44 0.10 |
| Ischemia duration | 1.97 | 1.40 | 1.41 | 0.202 | -1.34 5.28 |
| Reperfusion duration | -1.11 | 1.16 | -0.96 | 0.371 | -3.85 1.63 |
| Dosage | -1.18 | 1.61 | -0.73 | 0.487 | -4.98 2.62 |
| Treatment time | 0.51 | 1.60 | 0.32 | 0.760 | -3.27 4.29 |
| Rat species | -2.43 | 1.93 | -1.26 | 0.297 | -8.56 3.71 |
| Meta regression analysis on the results of MDA | | | | | |
| _ES | Coefficient | Std. err | t | p＞\|t\| | [95% conf. interval] |
| Gender distribution | 2.02 | 5.74 | 0.35 | 0.732 | -10.62 14.65 |
| Ischemia duration | -1.35 | 3.37 | -0.40 | 0.696 | -8.78 6.07 |
| Reperfusion duration | -.62 | 3.09 | -0.20 | 0.844 | -7.41 6.17 |
| Dosage | -1.73 | 2.78 | -0.62 | 0.546 | -7.85 4.392 |
| Route | -.148 | 2.37 | -0.06 | 0.951 | -5.36 5.06 |
| Treatment time | 1.67 | 3.11 | 0.54 | 0.603 | -5.19 8.52 |
| Rat species | 5.43 | 3.86 | 1.41 | 0.187 | -3.07 13.92 |
| Meta regression analysis on the results of LDH | | | | | |
| _ES | Coefficient | Std. err | t | p＞\|t\| | [95% conf. interval] |
| Gender distribution | -5.15 | 17.15 | -0.30 | 0.768 | -41.70 31.39 |
| Ischemia duration | -14.09 | 8.95 | -1.57 | 0.136 | -33.16 4.99 |
| Reperfusion duration | -15.56 | 6.85 | -2.27 | 0.038 | -30.17 -0.96 |
| Dosage | 2.25 | 5.10 | 0.44 | 0.666 | -8.62 13.11 |
| Route | 12.01 | 5.27 | 2.28 | 0.038 | 0.78 23.24 |
| Treatment time | -1.29 | 2.78 | -0.46 | 0.649 | -7.22 4.64 |
| Experiment type | 5.27 | 8.89 | 0.59 | 0.562 | -13.67 24.22 |
| Rat species | 11.27 | 7.61 | 1.48 | 0.159 | -4.95 27.48 |
| Meta regression analysis on the results of CK-MB | | | | | |
| _ES | Coefficient | Std. err | t | p＞\|t\| | [95% conf. interval] |
| Gender distribution | -2.88 | 5.72 | -0.50 | 0.636 | -17.59 11.82 |
| Ischemia duration | 1.81 | 4.390 | 0.41 | 0.698 | -9.48 13.09 |
| Reperfusion duration | -.177 | 3.72 | -0.05 | 0.964 | -9.73 9.38 |
| Dosage | -6.51 | 3.47 | -1.87 | 0.120 | -15.44 2.42 |
| Route | -1.64 | 2.65 | -0.62 | 0.564 | -8.46 5.18 |
| Treatment time | 1.27 | 4.05 | 0.31 | 0.767 | -9.15 11.68 |
| Rat species | 3.48 | 3.66 | 0.95 | 0.385 | -5.92 12.88 |
| Meta regression analysis on the results of myocardial infarct size | | | | | |
| _ES | Coefficient | Std. err | t | p＞\|t\| | [95% conf. interval] |
| Gender distribution | 7.01 | 7.07 | 0.99 | 0.341 | -8.40 22.41 |
| Ischemia duration | 0.40 | 6.36 | 0.06 | 0.951 | -13.47 14.26384 |
| Reperfusion duration | 0.82 | 3.78 | 0.22 | 0.833 | -7.43 9.06 |
| Dosage | 1.84 | 2.68 | 0.69 | 0.506 | -4.01 7.68 |
| Route | -0.48 | 2.45 | -0.19 | 0.849 | -5.82 4.87 |
| Treatment time | 0.34 | 2.13 | 0.16 | 0.876 | -4.31 4.99 |
| Experiment type | 5.47 | 7.22 | 0.76 | 0.463 | -10.26 21.20 |
| Rat species | 5.64 | 5.05 | 1.12 | 0.286 | -5.36 16.64 |
