# Supplementary material for: Efficacy of trimetazidine for myocardial ischemia-reperfusion injury in rat models: a systematic review and meta-analysis
Source: PeerJ. 2025 Jun 6;13:e19515. doi: 10.7717/peerj.19515 (PMC12147767; doi:10.7717/peerj.19515)
Supplement: Supplemental Information 6 [file peerj-13-19515-s006.docx]

**TABLE S5.** Subgroup analysis of SOD based on gender distribution, ischemia duration, reperfusion duration, dosage, and treatment time.

| **Criteria for grouping** | **Subgroup** | **n** | **Mean difference** | **Heterogeneity** | **Overall effect test** |
| --- | --- | --- | --- | --- | --- |
| Gender distribution | Male | 3 | 42.93 [26.34, 59.52] | Tau^2^ = 169.65; Chi^2^ = 14.34, df = 2 (P = 0.0008); I^2^ = 86% | Z = 5.07 (P < 0.001) |
|  | Male and Female | 2 | 25.71 [11.99, 39.42] | Tau^2^ = 0.00; Chi^2^ =0.03, df = 1 (P = 0.87); I^2^ = 0% | Z = 3.67 (P = 0.0002) |
| Ischemia duration | Time < 40min | 3 | 26.74 [18.63, 34.84] | Tau^2^ =0.00; Chi^2^ =0.06, df = 2 (P = 0.097); I^2^ =0% | Z = 6.47 (P < 0.001) |
|  | 40min ≤ Time ≤ 90min | 2 | 55.44 [18.74, 92.15] | Tau^2^ = 632.22; Chi^2^ = 9.31, df = 1 (P = 0.002); I^2^ = 89% | Z = 2.96 (P = 0.003) |
| Reperfusion duration | 30min ≤ Time < 120min | 1 | 76.12 [52.45, 99.79] | Not applicable | Z = 6.30 (P < 0.001) |
|  | 120min ≤ Time < 180min | 3 | 34.10 [25.03, 43.17] | Tau ^2^ = 26.28; Chi^2^ = 2.98, df = 2 (P = 0.23); I^2^ = 33% | Z = 7.37 (P < 0.001) |
|  | 180min ≤ Time ≤ 480min | 1 | 27.29 [17.25, 37.33] | Not applicable | Z = 5.33 (P < 0.001) |
| Dosage | 10mg·kg^-1^·d^-1^ ≤ Dosage < 20mg·kg^-1^·d^-1^ | 3 | 32.54 [23.08, 42.00] | Tau^2^ = 39.74; Chi^2^ = 4.96, df = 2 (P = 0.08); I^2^ = 60% | Z = 6.74 (P < 0.001) |
|  | 20mg·kg^-1^·d^-1^ ≤ Dosage ≤ 540mg·kg^-1^·d^-1^ | 2 | 50.70[2.18, 99.21] | Tau^2^ = 1113.11; Chi^2^ = 10.85, df = 1 (P = 0.0010); I^2^ = 91% | Z = 2.05 (P = 0.04) |
| Treatment time | Prior to ischemia | 1 | 76.12 [52.45, 99.79] | Not applicable | Z = 6.30 (P < 0.001) |
|  | Prior to reperfusion | 4 | 31.87 [23.80, 39.95] | Tau^2^ = 31.68; Chi^2^ = 5.97, df = 3 (P = 0.11); I^2^ = 50% | Z = 7.74 (P < 0.001) |
| Rat species | SD | 3 | 42.93 [26.34, 59.52] | Tau^2^ = 169.65; Chi^2^ = 14.34, df = 2 (P = 0.0008); I^2^ = 86% | Z = 5.07 (P < 0.001) |
|  | Wistar | 2 | 25.71 [11.99, 39.42] | Tau^2^ = 0.00; Chi^2^ = 0.03, df = 1 (P = 0.87); I^2^ = 0% | Z = 3.67 (P = 0.0002) |
