# Supplementary material for: Efficacy of trimetazidine for myocardial ischemia-reperfusion injury in rat models: a systematic review and meta-analysis
Source: PeerJ. 2025 Jun 6;13:e19515. doi: 10.7717/peerj.19515 (PMC12147767; doi:10.7717/peerj.19515)
Supplement: Supplemental Information 7 [file peerj-13-19515-s007.docx]

**TABLE S6.** Subgroup analysis of MDA based on gender distribution, ischemia duration, reperfusion duration, dosage, route, and treatment time.

| **Criteria for grouping** | **Subgroup** | **n** | **Mean difference** | **Heterogeneity** | **Overall effect test** |
| --- | --- | --- | --- | --- | --- |
| Gender distribution    Ischemia duration | Male | 10 | -0.26 [-0.33, -0.20] | Tau^2^ = 0.00; Chi^2^ = 1813.50, df = 9 (P < 0.001); I^2^ = 100% | Z = 8.24 (P < 0.001) |
|  | Male and Female | 3 | -0.52 [-1.27, 0.22] | Tau^2^ = 0.31; Chi^2^ =7.51, df = 2 (P = 0.02); I^2^ = 73% | Z = 1.38 (P = 0.17) |
|  | Time < 40min | 10 | -0.30 [-0.38, -0.22] | Tau^2^ = 0.00; Chi^2^ = 1535.67, df = 9 (P < 0.001); I^2^ = 99% | Z = 7.19 (P < 0.001) |
|  | 40min ≤ Time ≤ 90min | 3 | -0.08[-0.13, -0.03] | Tau^2^ = 0.00; Chi^2^ = 201.57, df = 2 (P < 0.001); I^2^ = 99% | Z = 3.11 (P = 0.002) |
| Reperfusion duration | 30min ≤ Time < 120min | 4 | -4.66 [-6.61, -2.71] | Tau^2^ = 3.23; Chi^2^ = 186.40, df = 3 (P < 0.001); I^2^ = 98% | Z = 4.68 (P < 0.001) |
|  | 120min ≤ Time < 180min | 6 | -0.06 [-0.11, -0.00] | Tau^2^ = 0.00; Chi^2^ = 2920.92, df = 5 (P < 0.001); I^2^ = 100% | Z = 2.07 (P = 0.04) |
|  | 180min ≤ Time ≤ 480min | 3 | -0.94[-2.07, 0.19] | Tau^2^ = 0.98; Chi^2^ = 195.99, df = 2 (P < 0.001); I^2^ = 99% | Z = 1.64 (P = 0.10) |
| Dosage | 3mg·kg^-1^·d^-1^ ≤ Dosage < 10mg·kg^-1^·d^-1^ | 4 | -1.97[-3.15, -0.80] | Tau^2^ = 1.21; Chi^2^ = 243.91, df = 3 (P < 0.001); I^2^ = 99% | Z =3.30 (P = 0.0010) |
|  | 10mg·kg^-1^·d^-1^ ≤ Dosage < 20mg·kg^-1^·d^-1^ | 5 | -0.05 [-0.11, 0.01] | Tau^2^ = 0.00; Chi^2^ = 281.27, df = 4 (P < 0.001); I^2^ = 99% | Z = 1.62 (P = 0.11) |
|  | 20mg·kg^-1^·d^-1^ ≤ Dosage ≤ 540mg·kg^-1^·d^-1^ | 4 | -3.77[-6.10, -1.43] | Tau^2^ = 4.29; Chi^2^ = 372.84, df = 3 (P < 0.001); I^2^ = 99% | Z = 3.16 (P = 0.002) |
| Route | i.v | 4 | -0.77[-1.47, -0.07] | Tau^2^ =0.50; Chi^2^ = 199.38, df = 3 (P < 0.001); I^2^ = 98% | Z = 2.16 (P = 0.03) |
|  | i.g | 1 | -99.62 [-114.11, -85.13] | Not applicable | Z = 13.47 (P < 0.001) |
|  | i.p | 8 | -0.13 [-0.19, -0.07] | Tau^2^ =0.00; Chi^2^ = 1472.34, df = 7 (P < 0.001); I^2^ = 100% | Z = 4.29 (P < 0.001) |
| Treatment time | Prior to ischemia | 5 | -2.55 [-3.56, -1.54] | Tau^2^ = 0.97; Chi^2^ =268.86, df = 4 (P < 0.001); I^2^ = 99% | Z =4.95 (P < 0.001) |
|  | Prior to reperfusion | 6 | -0.03 [-0.05, 0.00] | Tau^2^ = 0.00; Chi^2^ = 278.77, df = 5 (P < 0.001); I^2^ = 98% | Z = 1.84 (P = 0.07) |
|  | During ischemia | 2 | -1.42 [-1.62, -1.22] | Tau^2^ = 0.00; Chi^2^ = 0.09, df = 1 (P = 0.77); I^2^ = 0% | Z = 14.10 (P < 0.001 |
| Rat species | SD | 6 | -0.08 [-0.13, -0.04] | Tau^2^ = 0.00; Chi^2^ = 3657.31, df = 5 (P < 0.001); I^2^ = 100% | Z = 3.47 (P = 0.0005) |
|  | Wistar | 7 | -2.26 [-3.15, -1.37] | Tau^2^ = 1.10; Chi^2^ = 156.09, df = 6 (P < 0.001); I^2^ = 96% | Z = 4.98 (P < 0.001) |
