# Supplementary material for: Efficacy of trimetazidine for myocardial ischemia-reperfusion injury in rat models: a systematic review and meta-analysis
Source: PeerJ. 2025 Jun 6;13:e19515. doi: 10.7717/peerj.19515 (PMC12147767; doi:10.7717/peerj.19515)
Supplement: Supplemental Information 8 [file peerj-13-19515-s008.docx]

**TABLE S7.** Subgroup analysis of LDH based on gender distribution, ischemia duration, reperfusion duration, dosage, route, treatment time, and experiment type.

| **Criteria for grouping** | **Subgroup** | **n** | **Mean difference** | **Heterogeneity** | **Overall effect test** |
| --- | --- | --- | --- | --- | --- |
| Gender distribution | Male | 16 | -18.75 [-20.09, -17.41] | Tau^2^ = 2.00; Chi^2^ = 3741.37, df = 15 (P < 0.001); I^2^ = 100% | Z = 27.37 (P < 0.001) |
|  | Female | 1 | -148.00 [-176.51, -119.49] | Not applicable | Z = 10.17 (P < 0.001) |
| Ischemia duration | Time < 40min | 13 | -46.10 [-60.69, -31.51] | Tau^2^ = 593.89; Chi^2^ = 769.14, df = 12 (P < 0.001); I^2^ = 98% | Z = 6.19 (P < 0.001) |
|  | 40min ≤ Time ≤ 90min | 4 | -28.72 [-49.92, -7.52] | Tau^2^ = 382.98, Chi^2^ = 1583.21, df = 3 (P < 0.001); I^2^ = 100% | Z = 2.66 (P = 0.008) |
| Reperfusion duration | 30min ≤ Time < 120min | 4 | -33.77 [-54.84, -12.70] | Tau^2^ = 353.86; Chi^2^ = 40.01, df =3 (P < 0.001); I^2^ = 93% | Z = 3.14 (P = 0.002) |
|  | 120min ≤ Time < 180min | 12 | -48.57[-62.76, -34.38] | Tau^2^ = 506.25; Chi^2^ = 2347.45, df =11 (P < 0.001); I^2^ = 100% | Z = 6.71 (P < 0.001) |
|  | 180min ≤ Time ≤ 480min | 1 | -4.43 [-4.47, -4.39] | Not applicable | Z = 204.78 (P<0.001) |
| Dosage | 10mg·kg^-1^·d^-1^ ≤ Dosage < 20mg·kg^-1^·d^-1^ | 6 | -33.36 [-45.96, -20.76] | Tau^2^ = 179.05; Chi^2^ = 480.43, df = 5 (P < 0.001); I^2^ = 99% | Z = 5.19 (P < 0.001) |
|  | 20mg·kg^-1^·d^-1^ ≤ Dosage ≤ 540mg·kg^-1^·d^-1^ | 6 | -53.49[-82.75, -24.24] | Tau^2^ = 1152.29; Chi^2^ = 2441.88, df = 5 (P < 0.001); I^2^ = 99% | Z =3.58 (P = 0.0003) |
|  | 10μmol·L^-1^≤Dosage≤50μmol·L^-1^ | 5 | -36.75[-56.32, -17.18] | Tau^2^ = 410.10; Chi^2^ = 233.23, df = 4 (P < 0.001); I^2^ = 98% | Z = 3.68 (P = 0.0002) |
| Route | i.p | 3 | -5.18 [-6.38, -3.97] | Tau^2^ = 0.79; Chi^2^ = 1473.40, df = 2 (P < 0.001); I^2^ = 100% | Z = 8.43 (P P<0.001) |
|  | i.g | 9 | -58.97 [-86.50, -31.43] | Tau^2^ = 1538.52; Chi^2^ = 322.90, df = 8 (P < 0.001); I^2^ = 98% | Z = 4.20 (P < 0.001) |
|  | ecp | 5 | -36.75 [-56.32, -17.18] | Tau^2^ = 410.10; Chi^2^ = 233.33, df = 4 (P < 0.001); I^2^ = 98% | Z = 3.68 (P = 0.0002) |
| Treatment time | Prior to ischemia | 7 | -63.60 [-113.05, -14.16] | Tau^2^ = 3980.04; Chi^2^ =296.04, df = 6 (P < 0.001); I^2^ = 98% | Z = 2.52 (P = 0.01) |
|  | Prior to reperfusion | 5 | -36.82 [-54.86, -18.78] | Tau^2^ = 389.48; Chi^2^ = 150.98, df = 4 (P < 0.001); I^2^ = 97% | Z = 4.00 (P < 0.001) |
|  | After reperfusion | 2 | -16.80 [-57.44, 23.84] | Tau^2^ = 582.85; Chi^2^ = 2.10, df = 1 (P = 0.15); I^2^ = 52% | Z = 0.81 (P = 0.42) |
|  | During ischemia and reperfusion | 1 | -14.00[-17.39, -10.61] | Not applicable | Z = 8.08 (P < 0.001) |
|  | During reperfusion | 1 | -42.14[-52.99, -31.29] | Not applicable | Z = 7.61 (P < 0.001) |
|  | Prior to ischemia and prior to reperfusion | 1 | -45.59[-47.59, -43.19] | Not applicable | Z = 40.39 (P < 0.001) |
| Experiment type | In vivo | 12 | -11.15 [-12.42, -9.88] | Tau^2^ = 1.21; Chi^2^ = 2261.33, df = 11 (P < 0.001); I^2^ = 100% | Z = 17.19 (P < 0.001) |
|  | Ex vivo | 5 | -36.75 [-56.32, -17.18] | Tau^2^ =410.10; Chi^2^ = 233.33, df = 4 (P < 0.001); I^2^ = 98% | Z = 3.68 (P = 0.0002) |
| Rat species | SD | 11 | -116.22 [-128.42, -104.01] | Tau^2^ = 199.46; Chi^2^ = 3951.42, df = 10 (P < 0.001); I^2^ = 100% | Z = 18.67 (P < 0.001) |
|  | Wistar | 6 | -104.79 [-164.97, -44.60] | Tau^2^ = 2977.78; Chi^2^ = 123.62, df = 5 (P < 0.001); I^2^ = 96% | Z = 3.41 (P = 0.0006) |
