# Supplementary material for: Efficacy of trimetazidine for myocardial ischemia-reperfusion injury in rat models: a systematic review and meta-analysis
Source: PeerJ. 2025 Jun 6;13:e19515. doi: 10.7717/peerj.19515 (PMC12147767; doi:10.7717/peerj.19515)
Supplement: Supplemental Information 9 [file peerj-13-19515-s009.docx]

**TABLE S8.** Subgroup analysis of CK-MB based on gender distribution, ischemia duration, reperfusion duration, dosage, route, and treatment time.

| **Criteria for grouping** | **Subgroup** | **n** | **Mean difference** | **Heterogeneity** | **Overall effect test** |
| --- | --- | --- | --- | --- | --- |
| Gender distribution | Male | 6 | -0.66 [-1.09, -0.23] | Tau^2^ = 0.23; Chi^2^ =554.16, df = 5 (P < 0.001); I^2^ = 99% | Z = 3.00 (P = 0.003) |
|  | Female | 1 | -121.00[-137.66, -104.34] | Not applicable | Z = 14.24 (P < 0.001) |
| Ischemia duration | Time < 40min | 5 | -0.51 [-1.04, 0.02] | Tau^2^ = 0.28; Chi^2^ = 650.73, df = 4 (P < 0.001); I^2^ = 99% | Z = 1.87 (P = 0.06) |
|  | 40min ≤ Time ≤ 90min | 2 | -8.92 [-32.82, 14.99] | Tau^2^ = 199.98; Chi^2^ = 2.05, df = 1 (P = 0.15); I^2^ = 51% | Z = 0.73 (P = 0.46) |
| Reperfusion duration | 30min ≤ Time < 120min | 1 | -0.05 [-0.23, 0.13] | Not applicable | Z = 0.53 (P = 0.59) |
|  | 120min ≤ Time < 180min | 5 | -1.13 [-1.79, -0.47] | Tau^2^ = 0.33; Chi^2^ = 736.05, df = 4 (P < 0.001); I^2^ = 99% | Z = 3.36 (P = 0.0008) |
|  | 180min ≤ Time ≤ 480min | 1 | -0.40[-0.82, 0.02] | Not applicable | Z = 1.86 (P = 0.06) |
| Dosage | 10mg·kg^-1^·d^-1^ ≤ Dosage < 20mg·kg^-1^·d^-1^ | 5 | -2.35 [-4.28, -0.43] | Tau^2^ = 2.91; Chi^2^ = 317.84, df = 4 (P < 0.001); I^2^ = 99% | Z = 2.40 (P = 0.02) |
|  | 20mg·kg^-1^·d^-1^ ≤ Dosage ≤ 540mg·kg^-1^·d^-1^ | 2 | -0.54 [-1.17, 0.09] | Tau^2^ = 0.20; Chi^2^ = 431.92, df = 1 (P < 0.001); I^2^ = 100% | Z = 1.69 (P = 0.09) |
| Route | i.v | 1 | -0.05 [-0.23, 0.13] | Not applicable | Z = 0.53 (P = 0.59) |
|  | i.p | 2 | -60.31 [-178.67, 58.05] | Tau^2^ = 7257.78; Chi^2^ = 201.91, df = 1 (P < 0.001); I^2^ = 100% | Z = 1.00 (P = 0.32) |
|  | i.g | 4 | -1.03[-1.69, -0.36] | Tau^2^ = 0.33; Chi^2^ = 53.83, df = 3 (P < 0.001); I^2^ = 94% | Z = 3.03 (P = 0.002) |
| Treatment time | Prior to ischemia | 4 | -1.54 [-3.09, 0.02] | Tau^2^ = 1.27; Chi^2^ = 271.55, df = 3 (P < 0.001); I^2^ = 99% | Z = 1.94 (P = 0.05) |
|  | Prior to reperfusion | 3 | -0.79 [-1.86, 0.27] | Tau^2^ = 0.86; Chi^2^ = 138.69, df = 2 (P< 0.001); I^2^ = 99% | Z = 1.46 (P = 0.14) |
| Rat species | SD | 4 | -8.15[-13.04, -3.25] | Tau^2^ = 23.44; Chi^2^ = 481.92, df = 3 (P < 0.001); I^2^ = 99% | Z = 3.26 (P = 0.001) |
|  | Wistar | 3 | -503.63 [-1402.41, 395.15] | Tau^2^ = 616183.50; Chi^2^ = 204.94, df = 2 (P < 0.001); I^2^ = 99% | Z = 1.10 (P = 0.27) |
