# Supplementary material for: Efficacy of trimetazidine for myocardial ischemia-reperfusion injury in rat models: a systematic review and meta-analysis
Source: PeerJ. 2025 Jun 6;13:e19515. doi: 10.7717/peerj.19515 (PMC12147767; doi:10.7717/peerj.19515)
Supplement: Supplemental Information 10 [file peerj-13-19515-s010.docx]

**TABLE S9.** Subgroup analysis of myocardial infarct size based on gender distribution, ischemia duration, reperfusion duration, dosage, route, treatment time, and experiment type.

| **Criteria for grouping** | **Subgroup** | **n** | **Mean difference (MD)** | **Heterogeneity** | **Overall effect test** |
| --- | --- | --- | --- | --- | --- |
| Gender distribution | Male | 12 | -12.42 [-15.67, -9.17] | Tau^2^ = 30.76; Chi^2^ = 1570.48, df = 11 (P<0.001); I^2^ = 99% | Z = 7.49 (P < 0.001) |
|  | Male and Female | 2 | -11.67 [-16.22, -7.11] | Tau^2^ = 0.00; Chi^2^ = 0.12, df = 1 (P = 0.73); I^2^ = 0% | Z = 5.02 (P < 0.001) |
| Ischemia duration | Time < 40min | 11 | -11.27 [-15.39, -7.16] | Tau^2^ = 45.29; Chi^2^ =1465.74, df = 10 (P < 0.001); I^2^ = 99% | Z = 5.37 (P < 0.001) |
|  | 40min ≤ Time ≤ 90min | 3 | -16.41 [-24.75, -8.08] | Tau^2^ = 50.02; Chi^2^ = 32.19, df = 2 (P < 0.001); I^2^ = 94% | Z = 3.86 (P = 0.0001) |
| Reperfusion duration | 30min ≤ Time < 120min | 3 | -10.21 [-16.28, -4.14] | Tau^2^ = 27.03; Chi^2^ = 230.24, df = 2 (P < 0.001); I^2^ = 99% | Z = 3.30 (P = 0.0010) |
|  | 120min ≤ Time < 180min | 7 | -14.14[-20.08, -8.20] | Tau^2^ = 58.31; Chi^2^ = 131.65, df = 6 (P < 0.001); I^2^ = 95% | Z = 4.67 (P < 0.001) |
|  | 180min ≤ Time ≤ 480min | 4 | -11.15 [-17.62, -4.67] | Tau^2^ = 43.22; Chi^2^ = 508.82, df = 3 (P < 0.001); I^2^ = 99% | Z = 3.37 (P = 0.0007) |
| Dosage | 3mg·kg^-1^·d^-1^ ≤ Dosage < 10mg·kg^-1^·d^-1^ | 3 | -15.35 [-15.82, -14.89] | Tau^2^ = 0.00; Chi^2^ = 1.14, df = 2 (P = 0.56); I^2^ = 0% | Z = 64.79 (P <0.001) |
|  | 10mg·kg^-1^·d^-1^ ≤ Dosage < 20mg·kg^-1^·d^-1^ | 5 | -6.70[-10.97, -2.43] | Tau^2^ = 20.44; Chi^2^ = 121.33, df = 4 (P < 0.001); I^2^ = 97% | Z = 3.07 (P = 0.002) |
|  | 20mg·kg^-1^·d^-1^ ≤ Dosage < 540mg·kg^-1^·d^-1^ | 4 | -14.36 [-19.90, -8.81] | Tau^2^ = 28.37; Chi^2^ = 522.36, df = 3 (P < 0.001); I^2^ = 99% | Z = 5.08 (P < 0.001) |
|  | 10μmol·L^-1^≤Dosage≤50μmol·L^-1^ | 2 | -17.20 [-37.27, 2.87] | Tau^2^ = 203.17; Chi^2^ = 32.03, df = 1 (P < 0.001); I^2^ = 97% | Z = 1.68 (P = 0.09) |
| Route | i.v | 4 | -13.67 [-17.99, -9.35] | Tau^2^ = 19.01; Chi^2^ =243.30, df = 3 (P < 0.001); I^2^ = 99% | Z = 6.20 (P < 0.001) |
|  | i.g | 7 | -10.30 [-19.46, -1.15] | Tau^2^ = 147.68; Chi^2^ =1176.56, df = 6 (P < 0.001); I^2^ = 99% | Z = 2.21 (P = 0.03) |
|  | i.p | 1 | -12.09 [-12.51, -11.67] | Not applicable | Z = 56.58 (P < 0.001) |
|  | ecp | 2 | -17.20 [-37.27, 2.87] | Tau^2^ = 203.17; Chi^2^ = 32.03, df = 1 (P < 0.001); I^2^ = 97% | Z = 1.68 (P = 0.09) |
| Treatment time | Prior to ischemia | 4 | -11.36 [-19.61, -3.10] | Tau^2^ = 68.17; Chi^2^ = 601.66, df = 3 (P < 0.001); I^2^ = 100% | Z =2.70 (P = 0.007) |
|  | Prior to reperfusion | 5 | -8.91 [-17.44, -0.38] | Tau^2^ = 90.12; Chi^2^= 667.15, df = 4 (P < 0.001); I^2^ = 99% | Z = 2.05 (P = 0.04) |
|  | After reperfusion | 2 | -12.09 [-12.51, -11.67] | Tau^2^ = 0.00; Chi^2^= 0.01, df = 1 (P = 0.92); I^2^ = 0% | Z = 56.79 (P < 0.001) |
|  | During ischemia | 2 | -15.90 [-17.01, -14.78] | Tau^2^ = 0.00; Chi^2^= 0.04, df = 1 (P = 0.84); I^2^ =0% | Z = 27.95 (P < 0.001) |
|  | Prior to ischemia and prior to reperfusion | 1 | -27.48 [-32.82, -22.14] | Not applicable | Z = 10.08 (P < 0.001) |
| Experiment type | In vivo | 12 | -11.63 [-14.88, -8.38] | Tau^2^ = 30.20; Chi^2^ = 1537.77, df = 11 (P < 0.001); I^2^ = 99% | Z = 7.02 (P < 0.001) |
|  | Ex vivo | 2 | -17.20 [-37.27, 2.87] | Tau^2^ = 203.17; Chi^2^ = 32.03, df = 1 (P < 0.001); I^2^ = 97% | Z = 1.68 (P = 0.09) |
| Rat species | SD | 7 | -12.53[-17.02, -8.05] | Tau^2^ = 34.55; Chi^2^ = 1386.55, df = 6 (P < 0.001); I^2^ = 100% | Z = 5.48 (P < 0.001) |
|  | Wistar | 7 | -11.31 [-15.21, -7.40] | Tau^2^ = 23.92; Chi^2^ = 141.55, df = 6 (P < 0.001); I^2^ = 96% | Z = 5.68 (P < 0.001) |
