# Supplementary material for: Efficacy of trimetazidine for myocardial ischemia-reperfusion injury in rat models: a systematic review and meta-analysis
Source: PeerJ. 2025 Jun 6;13:e19515. doi: 10.7717/peerj.19515 (PMC12147767; doi:10.7717/peerj.19515)
Supplement: Supplemental Information 13 [file peerj-13-19515-s013.pdf]

Animal review

A list of fields that can be edited in an update can be found here

1. * Review title.

Give the working title of the review. This must be in English. The title should have the interventions or exposures being reviewed and the associated health or social problems.

Efficacy of Trimetazidine for Myocardial Ischemia-Reperfusion Injury in Rats: A Meta-Analysis and Systematic Review

2. Original language title.

For reviews in languages other than English, this field should be used to enter the title in the language of the review. This will be displayed together with the English language title.

3. * Anticipated or actual start date.

Give the date when the systematic review commenced, or is expected to commence.

11/12/2022

4. * Anticipated completion date. [1 change]

Give the date by which the review is expected to be completed.

31/12/2024

5. * Stage of review at time of this submission.

Indicate the stage of progress of the review by ticking the relevant Started and Completed boxes. Additional information may be added in the free text box provided.

Please note: Reviews that have progressed beyond the point of completing data extraction at the time of initial registration are not eligible for inclusion in PROSPERO. Should evidence of incorrect status and/or completion date being supplied at the time of submission come to light, the content of the PROSPERO record will be removed leaving only the title and named contact details and a statement that inaccuracies in the stage of the review date had been identified.

This field should be updated when any amendments are made to a published record and on completion and publication of the review.

The review has not yet started: Yes

Review stage	Started	Completed
Preliminary searches	No	No
Piloting of the study selection process	No	No
Formal screening of search results against eligibility criteria	No	No
Data extraction	No	No
Risk of bias (quality) assessment	No	No
Data analysis	No	No

Provide any other relevant information about the stage of the review here.

6. * Named contact. [1 change]

The named contact acts as the guarantor for the accuracy of the information presented in the register record.

Xiaobin Zhang

Email salutation (e.g. "Dr Smith" or "Joanne") for correspondence:

Dr Zhang

7. * Named contact email.

Enter the electronic mail address of the named contact.

2021100056@sdutcm.edu.cn

8. * Named contact address.

Enter the full postal address for the named contact.

Shandong University of Traditional Chinese Medicine, Changqing District, Jinan City, Shandong Province, China

9. Named contact phone number

Enter the telephone number for the named contact, including international dialling code.

18353168255

10. * Organisational affiliation of the review.

Full title of the organisational affiliations for this review and website address if available. This field may be completed as ‘none’ if the review is not affiliated to any organisation.

Shandong University of Traditional Chinese Medicine

Organisation web address:

https://www.sdutcm.edu.cn/

11. * Review team members and their organisational affiliations. [1 change]

Give the personal details and the organisational affiliations of each member of the review team. Affiliation refers to groups or organisations to which review team members belong. **NOTE: email and country are now mandatory fields for each person.**

Dr Xiaobin Zhang. Shandong University of Traditional Chinese Medicine
Miss Zhanhui Duan. Shandong University of Traditional Chinese Medicine
Mrs Yanpu Yu. Department of Traditional Chinese Medicine External Treatment Center, Affiliated Hospital of Shandong University of Traditional Chinese Medicine
Mrs Chunjing Li. Shandong University of Traditional Chinese Medicine
Mrs Yujie Li. Shandong University of Traditional Chinese Medicine
Mr Mingyao Hao. Department of Traditional Chinese Medicine External Treatment Center, Affiliated Hospital of Shandong University of Traditional Chinese Medicine
Mrs Yuning Ma. Shandong University of Traditional Chinese Medicine
Professor Yuxia Ma. Shandong University of Traditional Chinese Medicine
Mrs Dongqing Du. Shandong University of Traditional Chinese Medicine

12. * Funding sources/sponsors.

Give details of the individuals, organisations, groups or other legal entities who take responsibility for initiating, managing, sponsoring and/or financing the review. Any unique identification numbers assigned to the review by the individuals or bodies listed should be included.

General project of national natural science foundation of China. Shandong Natural Science Foundation Joint Fund Project.

Grant number(s)

Grant number of 81774402. Grant number of ZR2021LZ044.

13. * Conflicts of interest.

List any conditions that could lead to actual or perceived undue influence on judgements concerning the main topic investigated in the review.

None

14. Collaborators.

Give the name, affiliation and role of any individuals or organisations who are working on the review but who are not listed as review team members.

15. * Review question.

Give details of the question to be addressed by the review, clearly and precisely.

Whether trimetazidine can increase superoxide dismutase (SOD) levels and decrease methane dicarboxylic aldehyde (MDA) levels in rats with myocardial ischemia-reperfusion injury.

Context and rationale

Provide a brief description of the context and rationale of the review, including information on the relevance of your review for human health (max 250 words).

Diseases of the cardiovascular system pose a serious threat to human health. myocardial ischemia-reperfusion injury is an important pathophysiological process following myocardial infarction, cardiopulmonary resuscitation and extracorporeal circulation procedures. Mitigating ischemia-reperfusion injury to the myocardium has been a hot topic of research. Myocardial ischemia-reperfusion injury occurs when, after a prolonged period of ischemia, the function and structure of the heart does not recover as blood perfusion is reestablished, but a more severe injury occurs. Trimetazidine is a drug for the treatment of ischaemic heart disease that increases the cells' tolerance to hypoxia and reduces myocardial damage. The therapeutic effects of trimetazidine on rats with myocardial ischemia-reperfusion injury were investigated by systematic evaluation using MDA and SOD as observation indicators, with a view to further revealing the exact mechanism of trimetazidine's intervention on myocardial ischemia-reperfusion injury and providing reference for subsequent studies and clinical use.

16. * Searches.

Give details of the sources to be searched, and any restrictions (e.g. language or publication period). The full search strategy is not required, but may be supplied as a link or attachment.

[illegible]

English and Chinese.

17. URL to search strategy.

Give a link to the search strategy or an example of a search strategy for a specific database if available (including the keywords that will be used in the search strategies).

Alternatively, upload your search strategy to CRD in pdf format. Please note that by doing so you are consenting to the file being made publicly accessible.

Do not make this file publicly available until the review is complete

18. * Human disease modelled. [1 change]

Give a short description of the disease, condition or healthcare domain being modelled.

Myocardial ischemia-reperfusion injury

19. * Animals/population. [1 change]

Give summary criteria for the animals being studied by the review, e.g. species, sex, details of disease model. Please include details of both inclusion and exclusion criteria.

Inclusion criteria:

?1?Rats as the animal model. (2) Myocardial ischemia-reperfusion injury as the disease model. (3) In vitro studies.

Exclusion criteria:

?1?The animal model is not Rats. (2) The disease model is not myocardial ischemia-reperfusion injury.(3) Ex vivo studies, studies in humans or in sillico studies.

20. * Intervention(s), exposure(s). [1 change]

Give full and clear descriptions of the nature of the interventions or the exposures to be reviewed (e.g. dosage, timing, frequency). Please include details of both inclusion and exclusion criteria.

Inclusion criteria:

Trimetazidine as the intervention.

Exclusion criteria:

Trimetazidine is not used as a treatment modality.

21. * Comparator(s)/control. [1 change]

Where relevant, give details of the type(s) of control interventions against which the experimental condition(s) will be compared (e.g. another intervention or a non-exposed control group). Please include details of both inclusion and exclusion criteria.

Inclusion criteria:

The intervention with trimetazidine was compared with normal saline or blank intervention. The dose and method of administration of termitazide, the duration of treatment and the follow-up period were not restricted.

Exclusion criteria:

(1) Not a randomized controlled study. (2) Trimetazidine is combined with other interventions.

22. * Study designs to be included. [1 change]

Give details of the study designs eligible for inclusion in the review. If there are no restrictions on the types of study design eligible for inclusion, or certain study types are excluded, this should be stated. Please include details of both inclusion and exclusion criteria.

Inclusion criteria:

Randomized controlled study.

Exclusion criteria:

Studies are clinical case reports and duplicates articles.

23. Other selection criteria or limitations applied. [1 change]

Give details of any other inclusion and exclusion criteria, e.g. publication types (reviews, conference abstracts), publication date, or language restrictions.

Studies that are reviews and irrelevant articles would be excluded. The only permitted languages are English and Chinese. The search period spanned from the launch of the individual databases to 20 December, 2022.

24. * Outcome measure(s).

Give detail of the outcome measures to be considered for inclusion in the review. Please include details of both inclusion and exclusion criteria.

Inclusion criteria:

superoxide dismutase (SOD)?methane dicarboxylic aldehyde (MDA).

Exclusion criteria:

None of superoxide dismutase (SOD) or formaldehyde (MDA) was assessed.

25. N/A.

This question does not apply to systematic reviews of animal studies for human health submissions.

26. * Study selection and data extraction. [1 change]

Procedure for study selection

Give the procedure for selecting studies for the review, including the screening phases (title and/or title-abstract and/or full-text), the number of researchers involved, and how discrepancies will be resolved.

The articles were selected from the relevant literature and two independent authors, who assessed the title,

abstract and full text, then selected relevant trials based on inclusion criteria. Areas of contention were resolved by discussion with a third person.

Prioritise the exclusion criteria

Multiple exclusion criteria may apply to an abstract/paper, which can cause discrepancies between reviewers in the reason for exclusion recorded. To avoid this, it is helpful to prioritize the exclusion criteria (e.g. 1) not an animal study; 2) not a myocardial infarction model, etc.) and record the highest ranking applicable criterion as the reason for exclusion. Please sort the exclusion criteria defined in questions 19 to 24. If applicable, do so for each screening phase.

(1.)The animals are not Rats.

(2.)The disease model is not myocardial ischemia-reperfusion injury.

(3.) Trimetazidine is not used as a treatment modality.

(4.) Trimetazidine is combined with other interventions.

(5.)Not a randomized controlled study.

(6.)Studies are clinical case reports, reviews, duplicates and irrelevant articles.

(7.)None of superoxide dismutase (SOD) or formaldehyde (MDA) was assessed.

Methods for data extraction

Describe methods for data extraction, including the number of reviewers performing data extraction, extraction of data from text and/or graphs, whether and how authors of eligible studies will be contacted to provide missing or additional data, etc.

Two reviewers independently performed the literature search, data extraction, and cross-checking. With the assistance of a third party, contentious matters were discussed and settled. Data will be extracted from text, table and/or graphs. In case of missing data, the reviewers will be contacted via the email address of the author in the article. By reading the titles, abstracts and specifics of the literature, the two authors extracted the following: name of first author, year of publication, type of study, myocardial ischemia-reperfusion model methodology, sample size, rat sex, age, weight, and interventions. Outcome measure included superoxide dismutase (SOD), methane dicarboxylic aldehyde (MDA).

Data to be extracted: study design

Specify the data to be extracted related to characteristics of the study design, e.g. controlled versus cross-over, number of experimental groups, etc.

Trimetazidine group versus control group.

Data to be extracted: animal model

Specify the data to be extracted related to characteristics of the animal model, e.g. species, sex of the animals, etc.

Sample size, species, age, weight, sex of the animals.

Data to be extracted: intervention of interest

Specify the data to be extracted related to characteristics of the intervention of interest, e.g. dose, timing, etc.

Trimetazidine therapeutic dose, administration time.

Data to be extracted: primary outcome(s)

Define the primary outcome measure(s). For each outcome measure, specify in which format data will be extracted, including the eligible units of measurement, and data type (continuous/dichotomous). A description of any other manipulation or transformation of the extracted data that is planned may be included.

Primary outcome was superoxide dismutase (SOD). The eligible unit of measurement was U/mol., and data type was continuous.

Data to be extracted: secondary outcome(s)

Define the secondary outcome measure(s). For each outcome measure, specify in which format data will be extracted, including the eligible units of measurement, and data type (continuous/dichotomous). A description of any other manipulation or transformation of the extracted data that is planned may be included.

Secondary outcome was methane dicarboxylic aldehyde (MDA). The eligible unit of measurement was nmol/ml, and data type was continuous.

Data to be extracted: other

Specify any other data or study characteristics to be extracted, e.g. bibliographical details, such as author, year and language.

Author, year.

21. * Risk of bias and/or quality assessment. [1 change]

State whether and how risk of bias and/or study quality will be assessed. Assessment tools specific for pre-clinical animal studies include SYRCLE's risk of bias tool and the CAMARADES checklist for study quality

No risk of bias and/or quality assessment planned

No

By use of SYRCLE’s risk of bias tool

Yes

By use of SYRCLE’s risk of bias tool adapted as follows:

No

By use of the CAMARADES checklist for study quality

No

By use of the CAMARADES checklist for study quality, adapted as follows:

No

Other criteria, namely

No

Method for risk of bias and/or quality assessment

Give the procedure for the risk of bias and/or quality assessment, including the number of reviewers involved, their contribution, and how discrepancies will be resolved.

Quality assessment of the included studies was conducted by two investigators independently using SYRCLE’s risk of bias tool, which contains domains evaluating sequence generation, baseline characteristics, allocation concealment, random housing, blinding, random and selective outcome assessments, incomplete outcome data, and other sources of bias. Any discrepancies concerning the assessments were resolved through the discussion with a methodological researcher.

28. * Strategy for data synthesis. [1 change]

Planned approach

For each outcome measure, specify whether a quantitative or narrative synthesis is planned and how this decision will be made.

Meta-analysis was performed for SOD, MDA . Standard mean difference (SMD) or mean difference (MD),

and 95% CI were used as effect analysis statistics. When the heterogeneity is small (P0.1, I² 50%), a fixed effect model is used; if the heterogeneity is large (P ? 0.1, I² ? 50%), a random effect model is used to combine statistics. Meta-analysis using the random-effects model will be conducted to pool RR. At least 5 articles were included in the meta-analysis.

If a meta-analysis is planned, please specify the following:

Effect measure

For each outcome measure, specify the effect measure to be used (e.g. mean difference, odds ratio etc.).

Mean difference.

Effect models

For each outcome measure, specify the statistical model of analysis (e.g. random-effects or fixed-effect model).

Random-effects model

Heterogeneity

Specify the statistical methods to assess heterogeneity (e.g. I², Q). For further guidance please refer to the introduction and practical guide to pre-clinical meta-analysis.

I²

Other

Specify other details of the meta-analysis methodology (e.g. correction for multiple testing, correction for multiple use of control group).

The Review Manager Version 5.3 software and stata 16.0 software were applied to perform statistical analyses.

21. * Analysis of subgroups or subsets. [1 change]

Subgroup analyses

Give any planned exploration of subgroups or subsets within the review. 'None planned' is a valid response if no subgroup analyses are planned.

Subgroup analysis was performed to assess the effects of different doses of trimetazidine on superoxide

dismutase (SOD) and methane dicarboxylic aldehyde (MDA).

Sensitivity

For each outcome measure, specify any sensitivity analyses you propose to perform.

Sensitivity analyses were performed to determine whether the findings were robust. The sensitivity analysis. If

of superoxide dismutase (SOD) and methane dicarboxylic aldehyde (MDA) were carried out respectively.

the heterogeneity is large, the source literature of heterogeneity can be excluded by sensitive analysis

Publication bias

Specify whether an assessment of publication bias is planned. If applicable, specify the method for assessment of publication bias.

Funnel plots and Egger's test

30. * Review type.

Type of review

Animal model review

No

Experimental animal exposure review

No

Pre-clinical animal intervention review

Yes

31. Language.

Select each country individually to add it to the list below, use the bin icon to remove any added in error.

English

There is not an English language summary

32. * Country.

Select the country in which the review is being carried out from the drop down list. For multi-national collaborations select all the countries involved.

China

33. Other registration details.

List other places where the systematic review protocol is registered. The name of the organisation and any unique identification number assigned to the review by that organisation should be included.

34. Reference and/or URL for published protocol.

Give the citation and link for the published protocol, if there is one.

Add web link to the published protocol.

Or, upload your published protocol here in pdf format. Note that the upload will be publicly accessible.

No I do not make this file publicly available until the review is complete

Please note that the information required in the PROSPERO registration form must be completed in full even if access to a protocol is given.

35. Dissemination plans.

Give brief details of plans for communicating essential messages from the review to the appropriate audiences.

No

Give brief details of plans for communicating review findings.?

36. * Keywords.

Give words or phrases that best describe the review. Separate keywords with a semicolon or new line.

Trimetazidine, myocardial ischemia-reperfusion injury, rats, meta-analysis.

37. Details of any existing review of the same topic by the same authors.

Give details of earlier versions of the systematic review if an update of an existing review is being registered, including full bibliographic reference if possible.

38. * Current review status.

Review status should be updated when the review is completed and when it is published.

Please provide anticipated publication date

Review_Ongoing

39. Any additional information.

Provide any further information the review team consider relevant to the registration of the review.

40. Details of final report/publication(s) or preprints if available.

This field should be left empty until details of the completed review are available OR you have a link to a

preprint. Give the full citation for the preprint or final report or publication of the systematic review.

Give the link to the published review or preprint.
